# A central mechanism of analgesia in mice and humans lacking the sodium channel Na_V_1.7

**DOI:** 10.1016/j.neuron.2021.03.012

**Published:** 2021-05-05

**Authors:** Donald Iain MacDonald, Shafaq Sikandar, Jan Weiss, Martina Pyrski, Ana P. Luiz, Queensta Millet, Edward C. Emery, Flavia Mancini, Gian D. Iannetti, Sascha R.A. Alles, Manuel Arcangeletti, Jing Zhao, James J. Cox, Robert M. Brownstone, Frank Zufall, John N. Wood

**Affiliations:** 1 Molecular Nociception Group, Wolfson Institute for Biomedical Research, University College London, Gower Street, London WC1E 6BT, UK; 2 Centre for Experimental Medicine & Rheumatology, Queen Mary University of London, Charterhouse Square, London EC1M 6BQ, UK; 3 Center for Integrative Physiology and Molecular Medicine, Saarland University, 66421 Homburg, Germany; 4 Department of Neuroscience, Physiology and Pharmacology, University College London, Gower Street, London WC1E 6BT, UK; 5 Neuroscience and Behaviour Laboratory, Istituto Italiano di Tecnologia, Rome, Italy; 6 UCL Queen Square Institute of Neurology, London WC1N 3BG, UK

**Keywords:** pain, analgesia, sodium channels, Na_V_1.7, human genetics, endogenous opioids, neurotransmitter release

## Abstract

Deletion of *SCN9A* encoding the voltage-gated sodium channel Na_V_1.7 in humans leads to profound pain insensitivity and anosmia. Conditional deletion of Na_V_1.7 in sensory neurons of mice also abolishes pain, suggesting that the locus of analgesia is the nociceptor. Here we demonstrate, using *in vivo* calcium imaging and extracellular recording, that Na_V_1.7 knockout mice have essentially normal nociceptor activity. However, synaptic transmission from nociceptor central terminals in the spinal cord is greatly reduced by an opioid-dependent mechanism. Analgesia is also reversed substantially by central but not peripheral application of opioid antagonists. In contrast, the lack of neurotransmitter release from olfactory sensory neurons is opioid independent. Male and female humans with Na_V_1.7-null mutations show naloxone-reversible analgesia. Thus, inhibition of neurotransmitter release is the principal mechanism of anosmia and analgesia in mouse and human Nav1.7-null mutants.

## Highlights

• Loss of sodium channel Na_V_1.7 abolishes pain without silencing peripheral nociceptors
• Synaptic input to dorsal horn is compromised by an opioid-dependent mechanism
• Impaired neurotransmission from olfactory sensory neurons is opioid independent
• Blocking opioid receptors reverses analgesia in mice and humans lacking Na_V_1.7

## Teaser

Loss of the peripheral sodium channel Na_V_1.7 causes profound insensitivity to pain. MacDonald et al. show that nociceptor activity is unaffected by Na_V_1.7 deletion but that synaptic input to the dorsal horn is compromised by an opioid-dependent mechanism. Blocking central opioid receptors reverses analgesia in mice and humans lacking Na_V_1.7.

## Introduction

Chronic pain afflicts a fifth of the population, but effective analgesics are few (Breivik et al., 2006). We urgently need new molecular targets to develop improved painkillers. One strategy is to identify genes involved in rare human monogenic pain disorders. Loss-of-function mutations in the gene *SCN9A*, encoding the voltage-gated sodium channel Na_V_1.7, lead to congenital insensitivity to pain (CIP) and anosmia, but innocuous sensation remains intact (Cox et al., 2006; Weiss et al., 2011). Gain-of-function mutations in *SCN9A* are associated with ongoing pain (Faber et al., 2012; Fertleman et al., 2006; Yang et al., 2004). Given the enriched expression of Na_V_1.7 in nociceptors, these discoveries point to a key role of Na_V_1.7 in controlling nociception in humans (Black et al., 2012). Because Na_V_1.7-null individuals are wholly pain free, this channel is a promising, human-validated drug target for pain relief.

Paradoxically, pharmacological blockade of the channel does not appear to be able to recapitulate the analgesia associated with functional deletion of the *SCN9A* gene (Emery et al., 2016a). It has been assumed that Na_V_1.7 plays a key role in action potential initiation in the peripheral nerve endings of nociceptors. However, neurogenic inflammation dependent on action potential propagation is not compromised in Na_V_1.7 nulls suggesting that peripheral terminals of sensory neurons are still functional in the absence of Na_v_1.7 (Gingras et al., 2014; McDermott et al., 2019; Vetter et al., 2017). Nonetheless, conditional knockout of *Scn9a* only in peripheral sensory neurons of mice reproduces the pain insensitivity of Na_V_1.7-null humans, affirming peripheral sensory neurons as the locus of analgesia. These animals show profound behavioral deficits in thermal, mechanical, inflammatory, and some forms of neuropathic pain (Minett et al., 2012, 2014)

Loss of Na_V_1.7 expression in peripheral sensory neurons leads to enhanced endogenous opioid peptide synthesis and potentiated opioid receptor function in sensory neurons (Isensee et al., 2017; Minett et al., 2015). A role of opioid signaling in Nav1.7-null analgesia has been confirmed through use of the opioid antagonist naloxone, which substantially reverses analgesia associated with channel deletion (Minett et al., 2015). Reversal of analgesia as a result of combined deletion of μ and δ opioid receptors to the same level as that achieved with naloxone provides further support for an important contribution of endogenous opioid signaling to Na_V_1.7-null-mediated analgesia (Pereira et al., 2018). In this study, we addressed the mechanism of Na_V_1.7-null analgesia, using complementary optical, electrophysiological, and pharmacological methods to study nociceptor function *in vivo* in mice and humans lacking Na_V_1.7.

## Results

### Deletion of Na_V_1.7 in sensory neurons decreases pain sensitivity without silencing peripheral nociceptors

We deleted *Scn9a* encoding Na_V_1.7 in peripheral sensory neurons of mice. Advillin-Cre or Wnt1-Cre was used to excise the floxed *Scn9a* allele, resulting in knockout of Na_V_1.7 restricted to sensory neurons or neural crest-derived neurons, respectively (Minett et al., 2012). We performed whole-cell voltage-clamp recordings of voltage-gated sodium currents in cultured sensory neurons from control animals homozygous for the floxed *Scn9a* allele, here called wild type (WT). Application of the Na_V_1.7-specific antagonist PF-05089771 (PF-771, 100 nM for 5 min) showed that ∼50% of the peak sodium current in medium-diameter nociceptor-like neurons can be attributed to Na_V_1.7 (Figures S1A and S1B; Alexandrou et al., 2016). In contrast, PF-771 had no effect on voltage-gated sodium currents recorded in sensory neurons from Advillin-Cre Na_V_1.7 knockout (KO^Adv^) or Wnt1-Cre Na_V_1.7 knockout (KO^Wnt^) mice, confirming functional loss of the channel (Figures S1C and S1D). In common with human Na_V_1.7-null individuals, both lines of conditional Na_V_1.7 KOs showed a profound analgesic phenotype characterized by increased withdrawal thresholds to noxious thermal and mechanical stimuli but intact responses to innocuous cold and tactile stimuli (Figures S2A–S2D).

**Figure 1:**
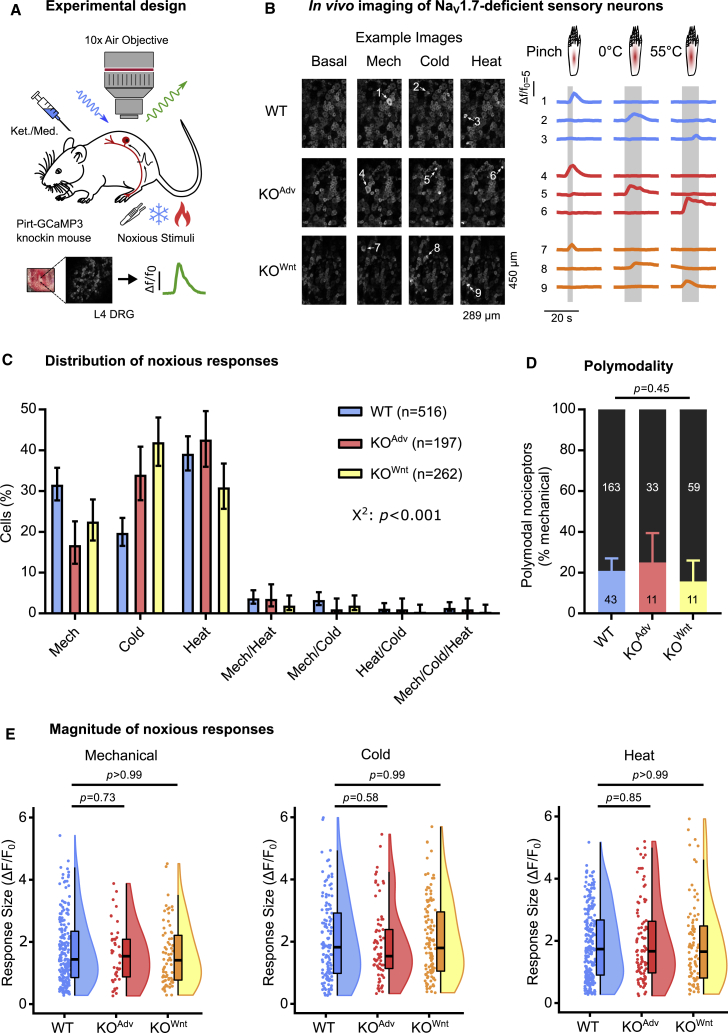
Na_V_1.7-deficient sensory neurons respond to noxious stimuli at the level of the soma *in vivo* (A) Schematic of the *in vivo* DRG imaging setup. (B) Example images and traces showing sensory neurons respond to noxious mechanical and thermal stimuli in WT and both Na_V_1.7 KO mouse lines. Each numbered trace corresponds to one cell. The data in this figure were obtained from 19 WT, 8 KO^Adv^, and 11 KO^Wnt^ animals. (C) Bar plot summarizing the distribution of all sensory neurons that responded to different noxious stimuli in WT and Na_V_1.7 KO animals. The error bars represent 95% confidence intervals, and proportions were compared using a chi-square test. n = 516 cells from WT (blue), n = 197 cells from KO^Adv^ (red), and n = 262 cells from KO^Wnt^ (yellow). (D) Bar plot showing a similar prevalence of polymodal nociceptors in WT and Na_V_1.7 KO mice. Polymodal nociceptors are defined as pinch-sensitive neurons that respond to any noxious thermal stimulus (color) and are expressed as a fraction of mechanically sensitive cells (black). The error bars represent 95% confidence intervals, and proportions were compared using the chi-square test. n = 206 cells from WT, n = 44 cells from KO^Adv^, and n = 70 cells from KO^Wnt^. (E ) Raincloud plots showing similar peak calcium responses (ΔF/F_0_) evoked by different noxious stimuli for WT and Na_V_1.7 KO lines. The mean response magnitude of KO lines was compared with the WT control using one-way ANOVA followed by post hoc Dunnett’s test. Mechanical: n = 206 cells from WT, n = 44 cells from KO^Adv^, and n = 70 cells from KO^Wnt^. Cold: n = 132 cells from WT, n = 73 cells from KO^Adv^, and n = 117 cells from KO^Wnt^. Heat: n = 234 cells from WT, n = 95 cells from KO^Adv^, and n = 88 cells from KO^Wnt^. See also Figures S1–S3.

To test the hypothesis that loss of Na_V_1.7 silences nociceptors, we monitored the responses of peripheral sensory neurons to noxious stimulation using *in vivo* calcium imaging (Emery et al., 2016b). We generated conditional Na_V_1.7 KO and WT mice on a Pirt-GCaMP3 background, where GCaMP3 is found in all peripheral sensory neurons (Kim et al., 2014). Using laser-scanning confocal microscopy, we imaged calcium signals in sensory neuron somata in the L4 dorsal root ganglia of live anesthetized animals (Figure 1A). Na_V_1.7-deficient sensory neurons readily responded to all noxious stimuli applied to the hindpaw (Figure 1B). We classified responding neurons into functionally defined cell types. Every cell type was present in WT and KO animals, but differences were apparent in the distribution of responses (Figure 1C). Fewer cells responded to noxious pinch in KO^Adv^ (44 of 197, 22%) and KO^Wnt^ (70 of 262, 27%) compared with WT (206 of 516, 40%) animals. This could be linked to loss of excitability in some nociceptors observed in *in vitro* culture experiments (Raouf et al., 2012). We observed a corresponding increase in the proportion of cold-sensing neurons in KO^Adv^ (73 of 197, 37%) and KO^Wnt^ (117 of 262, 45%) versus WT (132/516, 26%) animals. Importantly, the relative number of cells responding to noxious heat was not altered markedly between WT (234 of 516, 45%), KO^Adv^ (95 of 197, 48%), and KO^Wnt^ (88 of 262, 34%) lines despite behavioral insensitivity to heat (Figure S2C). We wondered whether polymodal nociceptors were silenced by loss of Na_V_1.7, here defined as pinch-sensitive cells that also respond to thermal stimuli. Polymodality did not differ between genotypes and was similar to previous reports, with 21% of WT, 25% of KO^Adv^, and 16% of KO^Wnt^ neurons categorized as polymodal (Figure 1D; Emery and Wood, 2019; Emery et al., 2016b; Lawson et al., 2019; Wang et al., 2018). Last, we measured the peak calcium signal (ΔF/F_0_) as a surrogate measure of single neuron excitability. When we quantified this for each stimulus type, there was no effect of Na_V_1.7 deletion on the maximum calcium responses (Figure 1E). Overall, although the distribution of cold and mechanical responses was altered, we found little evidence, using calcium imaging, of decreased nociceptor excitability in animals lacking Na_V_1.7, with no change in peak response magnitude to any stimulus, prevalence of polymodality, or number of noxious heat responses. Broadly similar results were obtained by calcium imaging of WT and KO^Adv^ mice virally expressing GCaMP6f (Figures S3A–S3D).

**Figure 2:**
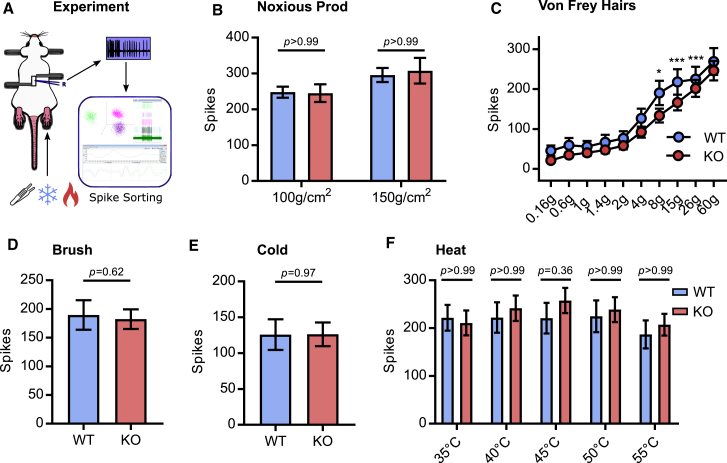
Excitability of Na_V_1.7-deficient sensory neurons *in vivo* (A) Schematic of the *in vivo* DRG extracellular recording setup. (B) Quantification of spikes fired in response to noxious prodding in WT (blue) and Na_V_1.7 KO (red). Data from Advillin-Cre and Wnt1-Cre Na_V_1.7 KO mice were pooled for these experiments. (C) Quantification of spikes fired in response to von Frey hair stimulation. For 8 g, p = 0.014. For 15 g, p < 0.001. For 26 g, p < 0.001. (D) Quantification of spikes fired in response to brushing. (E) Quantification of spikes fired in response to cooling. (F) Quantification of spikes fired in response to heating. For (B), (C), and (F), mean numbers of spikes fired in 10 s were compared using repeated-measures two-way ANOVA followed by post hoc Bonferroni test. For (D) and (E), means were compared using an unpaired t test. Error bars represent 95% confidence interval around the mean. n = 90 cells from 10 WT animals, and n = 146 cells from 13 KO^Adv/Wnt^ animals.

Although calcium imaging is ideally suited to monitoring population activity, we cannot directly measure action potential firing. We reasoned that this method may not be sensitive to subtler effects of Na_V_1.7 deletion on excitability. We therefore performed multi-unit extracellular recordings from dorsal root ganglia of live Na_V_1.7 KO and WT animals. For these experiments, we pooled data obtained from the KO^Adv^ and KO^Wnt^ lines, quantifying the number of action potentials fired in 10 s by polymodal afferents to peripheral stimuli of various modalities (Figure 2A). Firing evoked by noxious mechanical prodding was unchanged after deletion of Na_V_1.7 (Figure 2B). Intriguingly, although firing in response to most von Frey stimuli was normal, suprathreshold responses to 8-, 15-, and 26-g hairs showed a small reduction (Figure 2C). This is in spite of the normal hindpaw von Frey thresholds of Na_V_1.7 KO mice (Figure S2B). As expected, innocuous brush stimuli evoked action potentials equally well in WT and KO animals (Figure 2D). There was no appreciable change in the number of spikes triggered by ice water (Figure 2E) or noxious heat stimuli (Figure 2F). These data show that the diminished sensitivity of Na_V_1.7 KO mice to noxious heat or mechanical stimuli cannot be explained by decreased peripheral nociceptor excitability.

**Figure 3:**
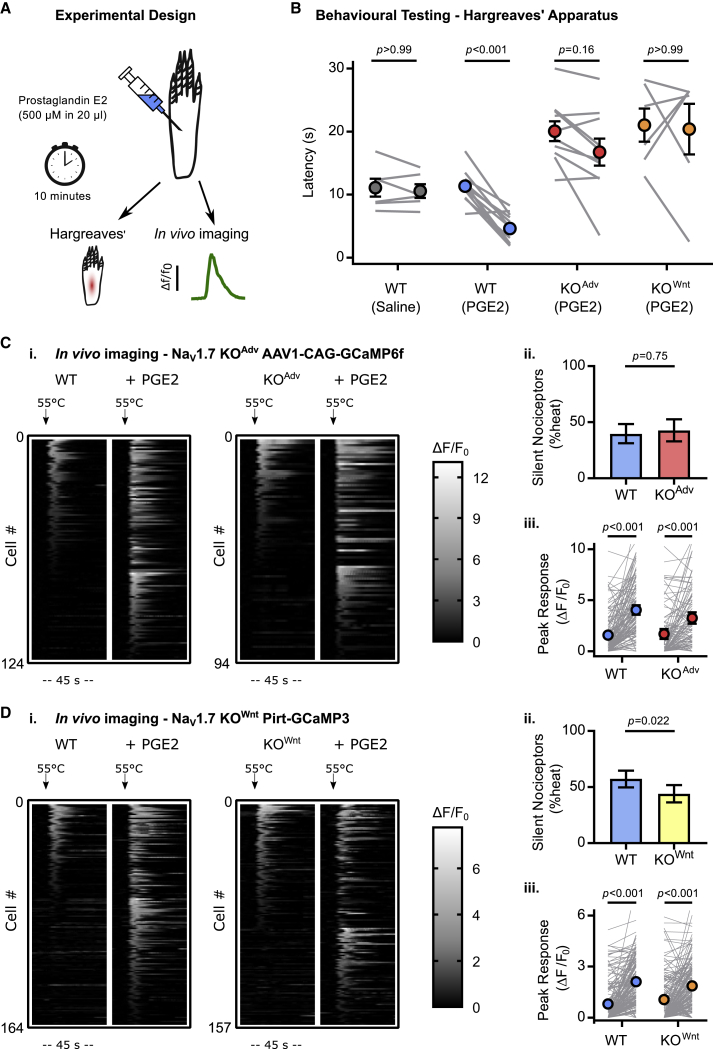
Na_V_1.7 deletion abolishes inflammatory pain without affecting peripheral sensitization (A) Schematic illustrating induction of acute inflammatory pain using PGE2. (B) Behavioral assessment of the effect of PGE2 on Hargreaves’ withdrawal latencies in WT and Na_V_1.7 KO animals, showing that KO mice do not develop heat hyperalgesia. The error bars represent standard error of the mean. Mean latencies before and after PGE2 were compared using repeated-measures two-way ANOVA followed by post hoc Sidak’s test. n = 6 animals for WT vehicle, n = 12 for WT PGE2, n = 10 for KO^Adv^ PGE2, and n = 6 for KO^Wnt^ PGE2. (C) Heatmaps (i) and quantification (ii and iii) showing unmasking of silent heat nociceptors by PGE2 in WT and Na_V_1.7 KO^Adv^ animals virally transduced with GCaMP6f. n = 124 cells from 4 WT animals, and n = 94 cells from 4 KO^Adv^. (D) Heatmaps (i) and quantification (ii and iii) showing unmasking of silent heat nociceptors by PGE2 in WT and Na_V_1.7 KO^Wnt^ animals expressing GCaMP3. n = 164 cells from 8 WT animals, and n = 157 cells from 11 KO^Wnt^ animals. For (C) and (D), the effect of genotype on PGE2-induced unmasking of silent nociceptors was compared using the chi-square test with Yates’ correction for proportions (i) and repeated-measures two-way ANOVA followed by post hoc Sidak’s test for mean response size (ii). Error bars represent 95% confidence intervals.

Of particular clinical interest is the fact that mice and humans lacking Na_V_1.7 show deficient inflammatory pain sensitization. Prostaglandin E2 (PGE2) is an important inflammatory mediator (Emery et al., 2016b). To investigate the mechanism by which Na_V_1.7 deletion impedes pain sensitization, we tested the effect of intraplantar injection of PGE2 (500 μM for 10 min) on whole-animal behavioral responses and sensory neuron calcium responses to noxious heat stimuli *in vivo* (Figure 3A). Both lines of conditional Na_V_1.7 KO mice failed to develop heat hyperalgesia (Figure 3B). In contrast, intraplantar injection of PGE2 unmasked silent nociceptors and increased heat responses in WT and KO^Adv^ animals virally expressing GCaMP6f, indicating that this form of peripheral sensitization is intact in mice lacking Na_V_1.7 (Figure 3C). We also observed robust sensitizing effects of PGE2 in KO^Wnt^ animals expressing GCaMP3, although the number of silent nociceptors activated was slightly less than WT (Figure 3D). Na_V_1.7 deletion therefore impairs thermal hyperalgesia but, paradoxically, does not abolish its physiological correlate: peripheral sensitization of nociceptors.

### Loss of Na_V_1.7 impairs synaptic transmission from nociceptors

The mechanism of Na_V_1.7 analgesia does not appear to arise from reduced peripheral excitability. We wondered whether the loss of function occurred at the nociceptor central terminal. To test this, we used the fluorescent glutamate sensor iGluSnFR to directly measure glutamate release from sensory afferent central terminals in spinal cord slices (Marvin et al., 2013). First, we virally transfected cultured dorsal root ganglion neurons with iGluSnFR. The iGluSnFR signal was present on the cell membrane and along neurites (Figure S4A). Bath application of a range of glutamate concentrations confirmed the sensitivity of the probe to extracellular glutamate concentrations within the physiological range (Figure S4B). We then virally transduced sensory neurons *in vivo* with iGluSnFR by intraperitoneal injection of AAV9-synapsin-iGluSnFR virus at post-natal day 2 (P2) (Figure S4C). Spinal cord slices were prepared for 2-photon imaging experiments at P9–P21. In the dorsal horn, we observed iGluSnFR fluorescence in the central processes of incoming afferents, but there was no evidence of iGluSnFR expression in spinal cord neuron somata (Figure S4D). In contrast, confocal imaging showed that iGluSnFR was widely expressed in the cell bodies of sensory neurons in dorsal root ganglia *in vivo*, confirming successful targeting of iGluSnFR to afferent neurons (Figure S4E).

**Figure 4:**
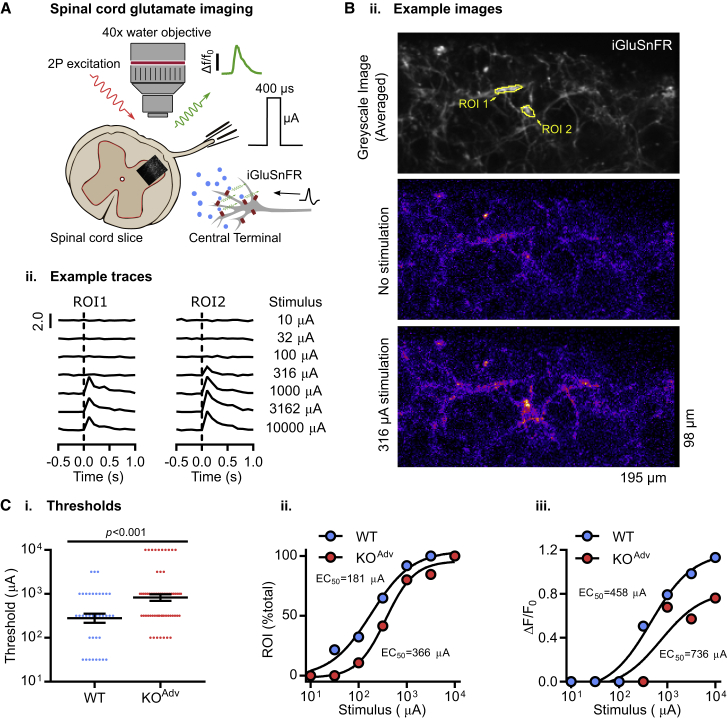
Decreased neurotransmitter release from the central terminals of Na_V_1.7-deficient sensory neurons (A) Schematic illustrating two-photon imaging of iGluSnFR-expressing afferent terminals in the dorsal horn of the spinal cord. iGluSnFR was virally expressed in sensory afferents. In horizontal slices, a suction electrode was used to electrically stimulate the attached dorsal root, driving glutamate release in lamina II of the dorsal horn. Increased extracellular glutamate at afferent terminals resulted in a time-locked increase in iGluSnFR fluorescence. (B) Example images of an area of lamina II dorsal horn in spinal cord slices from a Na_V_1.7 KO^Adv^ mouse expressing iGluSnFR in sensory afferent terminals (i). The top image is a greyscale average of the iGluSnFR signal over time, showing the afferent processes. The center image shows the signal in the absence of stimulation. In the bottom image, electrical stimulation causes localized increases in iGluSnFR fluorescence in discrete areas of the image. Two such areas are identified as regions of interest: region of interest (ROI) 1 and ROI 2. The images are pseudocolored to emphasize changes in fluorescence. Also shown are example traces of normalized increases in fluorescence (ΔF/F_0_) from each ROI to different single-pulse stimulation intensities applied to the dorsal root (ii). (C) Plots showing that the threshold current required to evoke glutamate release is increased in slices from KO^Adv^ mice. For (i), the mean absolute threshold was compared between genotypes using an unpaired t test. Error bars represent standard error of the mean. For (ii), WT EC_50_ = 181 μA, r^2^ = 0.99; KO^Adv^ EC_50_ = 366 μA, r^2^ = 0.99. For (iii), median evoked glutamate release (ΔF/F_0_) was compared between genotypes. WT EC_50_ = 458 μA, r^2^ = 0.98; KO^Adv^ EC_50_ = 736 μA, r^2^ = 0.86. n = 37 ROIs from 4 WT animals, and n = 65 ROIs from 6 KO^Adv^ mice. See also Figures S4 and S5.

To image synaptic transmission in the spinal cord, dorsal root stimulation was used to evoke neurotransmitter release from central afferent terminals expressing iGluSnFR in WT and KO^Adv^ mice (Figure 4A). We restricted our imaging of glutamate signals to lamina II in the dorsal horn of the spinal cord. Two-photon imaging at 10 Hz showed that glutamate release was readily evoked across a range of single-pulse stimulus intensities in dorsal horn of spinal cord slices, with iGluSnFR signals showing spatial localization to regions of interest (Figure 4B). Interestingly, the mean minimum stimulus current required to elicit release in slices from KO^Adv^ animals was 891 μA, 3-fold greater than the control WT value of 279 μA (Figure 4Ci). The EC_50_ current was also increased in KO^Adv^ slices, with a value of 366 μA compared with 181 μA in the WT (Figure 4Cii). In addition, the peak fluorescence change (ΔF/F_0_) was reduced in KO^Adv^ slices (Figure 4Ciii). These data are consistent with a reduction in glutamate release at the central terminal of sensory neurons in Na_V_1.7 KO^Adv^ animals.

Next we performed voltage-clamp recordings from lamina II neurons in spinal cord slices. We measured spontaneous excitatory postsynaptic currents (EPSCs), which tracks all excitatory input to the recorded neuron, including mono- and polysynaptic input from afferents expressing Na_V_1.7 (Kanellopoulos et al., 2018). There was a reduction in frequency but not amplitude, consistent with changes in presynaptic release (Figures S5A–S5C). These presynaptic deficits may account for the heightened pain thresholds of mice lacking Na_V_1.7 and associated diminished nociceptive input to the CNS (Minett et al., 2012).

### Opioid receptors are required for synaptic deficits in nociceptors but not olfactory sensory neurons lacking Na_V_1.7

**Figure 5:**
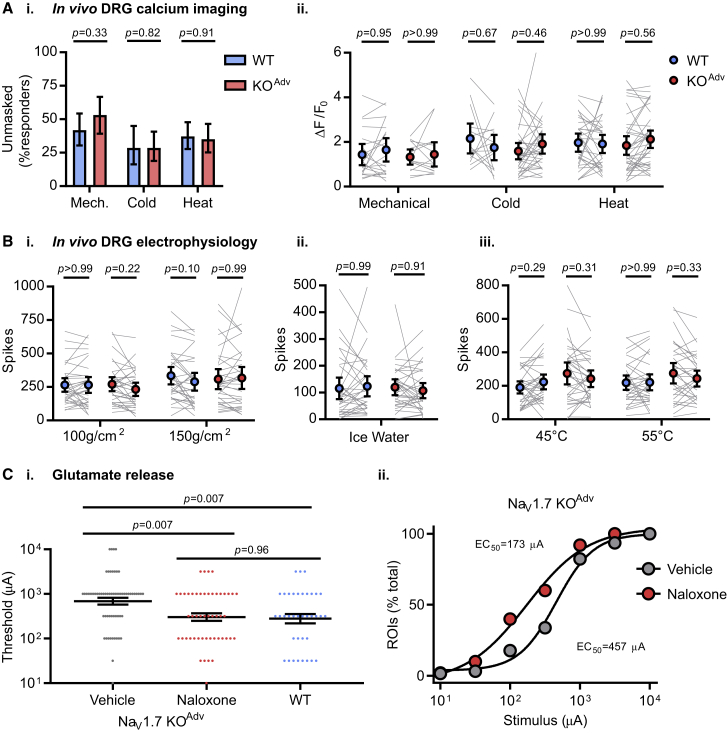
Opioid receptor blockade rescues impaired neurotransmission after Na_V_1.7 deletion but does not affect peripheral excitability (A) *In vivo* imaging of sensory neuron activity before and after treatment with systemic naloxone (2 mg/kg subcutaneously for 20 min) in WT (blue) and Na_V_1.7 KO^Adv^ (red) mice. Naloxone unmasked previously silent neurons in WT and KO^Adv^ mice (i). Proportions were compared using chi-square test with Yates’ correction. Naloxone had no effect on the peak calcium responses evoked by noxious stimuli in either genotype (ii). Mean peak calcium responses before and after naloxone were compared using repeated-measures two-way ANOVA followed by post hoc Sidak’s test. Data were obtained from 4 WT and 6 KO^Adv^ animals. Mechanical: n = 62 cells from WT, n = 47 cells from KO^Adv^. Cold: n = 35 cells from WT, n = 63 cells from KO^Adv^. Heat: n = 86 cells from WT, n = 74 cells from KO^Adv^. (B) *In vivo* extracellular recording of sensory neuron action potential firing before and after treatment with systemic naloxone (2 mg/kg subcutaneously for 20 min) in WT (blue) and Na_V_1.7 KO^Adv^ (red) mice. Naloxone had no effect on spiking evoked by noxious mechanical (i), ice water (ii) or heat (iii) stimuli. Mean spikes fired before and after naloxone were compared using repeated-measures two-way ANOVA followed by post hoc Sidak’s test. n = 33 from 6 WT animals, and n = 33 from 7 KO^Adv^ animals. (C) *Ex vivo* iGluSnFR imaging of glutamate release from sensory neuron central terminals in dorsal horn of spinal cord slices from 6 KO^Adv^ animals treated with vehicle (gray) or 100 μM naloxone (red). Naloxone reduced the mean threshold (i) and EC_50_ (ii) current required to elicit release to WT levels (blue). n = 62 ROIs for vehicle and n = 50 for naloxone in KO^Adv^. The WT data are the same as in Figure 4C (n = 37). Means were compared using one-way ANOVA followed by post hoc Tukey test. Error bars represent standard error of the mean.

We have shown previously that analgesia in mice and humans lacking Na_V_1.7 requires opioid receptors. This is due to upregulation of preproenkephalin (PENK) and enhanced opioid receptor signaling caused by Na_V_1.7 deletion (Isensee et al., 2017; Minett et al., 2015; Pereira et al., 2018). Does increased opioid signaling account for the synaptic deficits we observe in Na_V_1.7 KOs? We first investigated the effect of systemic naloxone injection *in vivo* (2 mg/kg subcutaneously for 20 min) on peripheral excitability of nociceptors. To our surprise, *in vivo* imaging experiments revealed that naloxone increased the number of responding cells in WT and KO^Adv^ mice, suggesting that tonic endogenous opioid activity is present in our preparation (Figure 5A). Importantly, however, the effect of naloxone on the number and size of responses was comparable across genotypes. Corroborating this, *in vivo* extracellular recording of sensory neurons from WT and KO^Adv^ animals found no effect of naloxone on action potential firing in response to noxious heat, cold, or mechanical stimuli (Figure 5B).

Because naloxone did not markedly affect peripheral excitability, we examined the relationship between opioid receptors and synaptic dysfunction in Na_V_1.7 KOs. Spinal cord slices from Na_V_1.7 KO^Adv^ animals were treated with vehicle or naloxone while measuring dorsal root stimulation-evoked glutamate release using iGluSnFR. Vehicle-treated KO^Adv^ slices showed a mean minimum stimulus current for eliciting release of 690 μA. This was reduced to 302 μA in naloxone-treated KO^Adv^ slices, comparable with the 279 μA threshold we observed previously in WT slices (Figure 5C). The EC_50_ current was 457 μA in the vehicle-treated group and decreased to 173 μA after naloxone, essentially identical to the WT value of 181 μA. These findings indicate that opioid receptor blockade reverses changes in glutamate release associated with deletion of Na_V_1.7.

**Figure 6:**
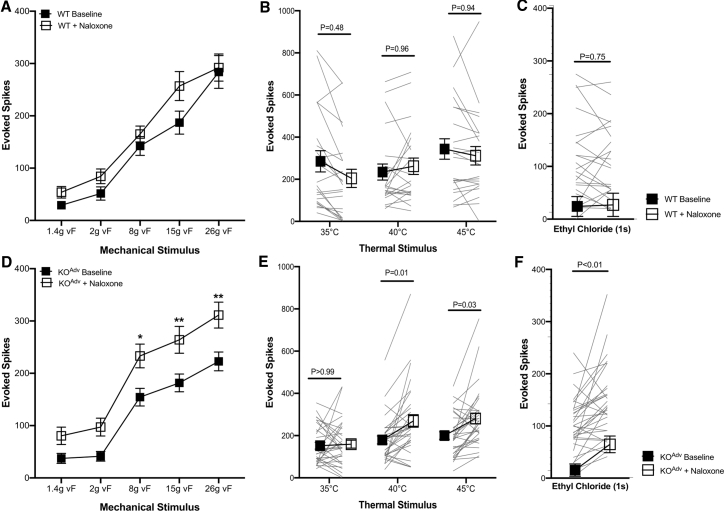
Impaired spinal sensory coding of noxious stimuli in peripheral Na_v_1.7 KO mice is reversed by opioid receptor blockade (A–F) Evoked activity of wide-dynamic-range deep dorsal horn neurons in WT and KO^Adv^ to (A and D) von Frey mechanical stimuli, (B and E) heat stimuli, and (C and F) noxious cold stimulation with ethyl chloride. Response profiles of 22 WDR neurons from WT mice (n = 7) and 32 WDR neurons from KO mice (n = 10) were recorded. Data are shown as mean number of action potentials fired ± SEM. ^∗^p < 0.05, ^∗∗^p < 0.01. All data were analyzed with two-way repeated-measures ANOVA with post hoc Sidak’s test (A, B, D, and E) and paired t test (C and F) with significance set at p > 0.05.

Next we tested whether these deficits in neurotransmitter release translated into a loss of noxious input into wide dynamic range (WDR) neurons of the spinal cord that could be reversed with naloxone. We investigated effects of systemic opioid receptor blockade on neural coding of lumbar spinal neurons following peripheral deletion of Na_v_1.7 using *in vivo* extracellular recordings in the deep dorsal horn of WT and KO^Adv^ mice. Mechanically evoked activity was assessed using punctate von Frey hairs (Figures 6A and 6D). Thermally evoked activity of spinal neurons was assessed using application of heat (Figure 6B and 6E) and noxious cold with ethyl chloride (Figures 6C and 6F). All stimuli were applied to the hindpaw peripheral receptive field of WDR neurons, and evoked action potentials over 10 s were recorded. WT and KO^Adv^ WDR neurons showed graded intensity coding to mechanical and heat stimulation. We then quantified changes in evoked firing and modality-based coding of spinal neurons of WT and KO^Adv^ mice following systemic administration of naloxone (2 mg/kg subcutaneously for 20 min). Following opioid receptor blockade, KO^Adv^ neurons showed augmented firing evoked by suprathreshold mechanical (8 g, p < 0.05; 15 g, p < 0.01; 26 g, p < 0.01), heat (40°C, p < 0.05; 45°C, p < 0.05), and cold (p < 0.01) simulation. We observed no significant difference in coding of spinal neurons of KO^Adv^ mice to innocuous intensities of mechanical and heat stimuli. Moreover, opioid receptor antagonism did not affect nociceptive response profiles of WT neurons to mechanical, thermal, or cold stimulation (p > 0.05 for all measures). These observations, coupled with earlier studies of opioid receptor-dependent analgesia and loss of neuropeptide release, highlight a significant deficit in neurotransmission that is opioid dependent in the absence of Na_V_1.7 (Minett et al., 2012, 2015).

**Figure 7:**
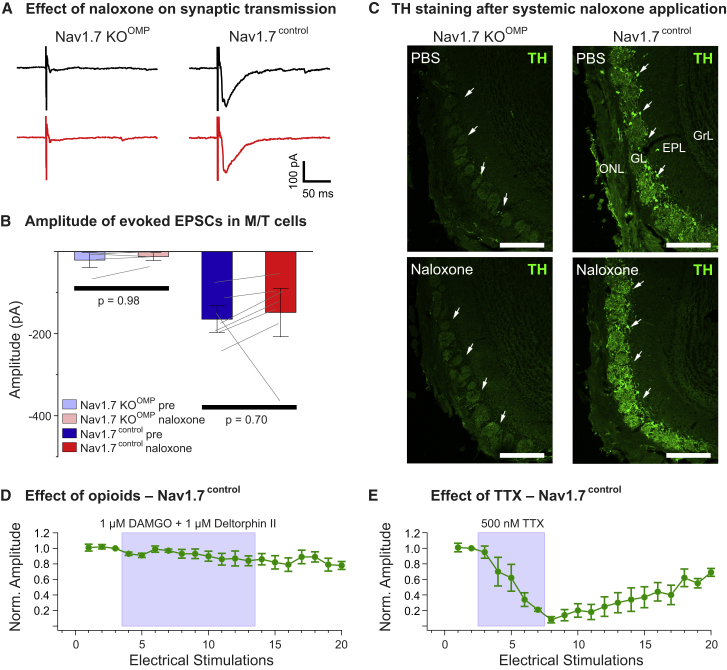
Opioid receptor blockade does not rescue synaptic transmission in mice lacking Na_V_1.7 in olfactory sensory neurons (A) Loss of synaptic transmission onto M/T cells in olfactory bulb slices of Na_V_1.7 KO^OMP^ mice after olfactory sensory neuron nerve stimulation cannot be rescued by 300 μM naloxone (left, red trace). Additionally, 300 μM naloxone does not affect M/T EPSCs to presynaptic nerve stimulation in control mice (right, red trace). (B) Summary plot showing that the morphine receptor antagonist naloxone (300 μM) does not affect EPSC amplitudes in M/T cells after presynaptic nerve stimulation in Na_V_1.7 KO^OMP^ (KO^OMP^ pre and naloxone, n = 17 from 5 animals) or in control mice (pre and naloxone, n = 7 from 4 animals). Error bars represent SEM. Means were compared using paired t test. (C) Confocal images of tyrosine hydroxylase (TH) immunostaining (green) in coronal cryosections of the main olfactory bulb (MOB) following systemic administration of PBS or naloxone of adult Na_V_1.7 KO^OMP^ (left) and control (right) mice. TH staining is absent in the glomerular layer (GL) of Na_V_1.7 KO^OMP^ mice independent of treatment (arrows), whereas the MOB of control mice shows robust TH labeling of neuronal processes and periglomerular cell somata (arrows). There is no difference in TH staining in control MOBs when comparing PBS versus naloxone administration. ONL, olfactory nerve layer; EPL, external plexiform layer; GrL, granule cell layer. Scale bars, 200 μm. (D) Time course showing that treatment with 1 μM DAMGO and 1 μM deltorphin II does not affect M/T cell EPSCs evoked by olfactory nerve stimulation (n = 4). Normalized EPSC peak amplitudes are plotted as a function of the number of electrical ONL stimulations (1-min intervals). Error bars represent SEM. (E) Time course showing that treatment with 500 nM TTX completely and reversibly abolishes M/T cell EPSCs evoked by ONL stimulation (n = 4). Error bars represent standard error of the mean.

Mice lacking Na_V_1.7 in olfactory sensory neurons (Nav1.7 KO^OMP^) are totally anosmic because of loss of transmitter release from olfactory nerve terminals (Weiss et al., 2011). To investigate whether these synaptic deficits are also dependent on opioid receptors, we recorded mitral/tufted (M/T) cells in horizontal olfactory bulb slices of KO^OMP^ mice. As expected, electrical stimulation (1 ms, 100 V) of the olfactory nerve layer harboring the olfactory sensory neuron axon terminals did not elicit a postsynaptic current in M/T cells of Nav1.7 KO^OMP^ mice (Figure 7A). To test whether the opioid receptor antagonist naloxone could restore transmitter release at this synapse, we bath-applied 300 μM naloxone for at least 10 min and recorded olfactory nerve-evoked responses in 17 M/T cells (5 animals). Naloxone did not lead to an increase in excitatory post-synaptic current (EPSC) amplitude (Figure 7B) (Nav1.7KO^OMP^ pre, −14 ± 0.4 pA; Na_V_1.7KO^OMP^ naloxone, −13.9 ± 0.03 pA). To exclude the possibility that naloxone affects transmitter release in general at this synapse, we additionally recorded M/T cells of control mice (Nav1.7^control^, 7 cells in 4 animals). Olfactory nerve stimulation caused activation of characteristic inward currents in M/T cells of control mice, with amplitudes of −165 ± 20 pA. Application of naloxone to the slice had only minor effects on evoked EPSCs (−148 ± 37 pA) (Figures 7A and 7B). As further corroboration, animals were injected intraperitoneally 3 times (30-min interval) with naloxone (2 mg/kg) or PBS and kept in the odor-rich environment of their home cage for the next 24 h. Animals were then sacrificed and perfused, and olfactory bulb coronal sections were stained against tyrosine hydroxylase (TH) protein. TH expression in olfactory bulb juxtaglomerular neurons is a correlate of afferent synaptic input because it requires odor-stimulated glutamate release from olfactory sensory neuron (OSN) terminals (Weiss et al., 2011). There was no obvious difference between PBS- and naloxone-treated animals in the amount of TH expression, with low to no expression of TH in KO^OMP^ mice and high expression in control mice (Figure 7C). These results are consistent with the finding that, in Nav1.7^control^ mice, treatment with a cocktail of opioid receptor agonists did not affect EPSCs evoked by olfactory nerve stimulation (Figure 7D). Tetrodotoxin (TTX), on the other hand, completely abolished synaptic transmission from OSNs to M/T cells (Figure 7E).

### Pain insensitivity of mice and humans lacking Na_V_1.7 depends on opioid signaling

**Figure 8:**
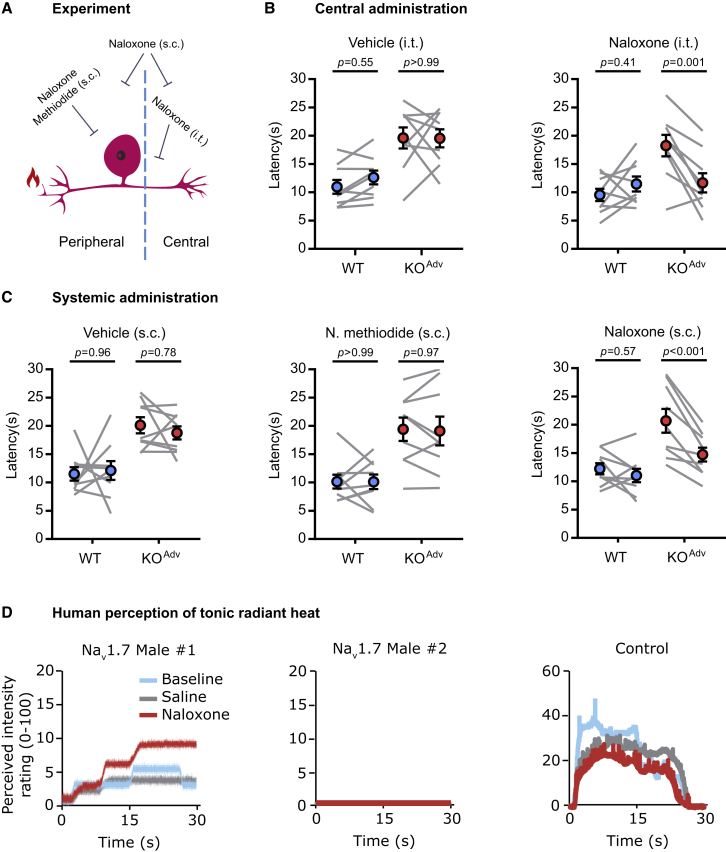
Blocking central opioid receptors reverses analgesia in mice and humans lacking Na_V_1.7 (A) Schematic of the behavioral pharmacology experiment. (B) Behavioral assessment of the effect of vehicle and the opioid receptor blocker naloxone (3 mM in 5 μL for 20 min) administered centrally by intrathecal injection. (C) Behavioral assessment of the effect of vehicle and the opioid receptor blockers naloxone (2 mg/kg for 20 min) and naloxone methiodide (N. methiodide; 2 mg/kg for 20 min) administered systemically by subcutaneous injection. N. methiodide is peripherally restricted and does not cross the blood-brain barrier. (D) Line plots showing the reported, perceived intensity of tonic, radiant heat stimuli (45°C–48°C) in two newly reported male Na_V_1.7-null individuals and one control participant, at baseline, during saline administration and after treatment with naloxone (12 mg). Naloxone appears to increase heat sensitivity in one Na_V_1.7-null participant (male 1), replicating previous observations in a single female null participant (Minett et al., 2015). Naloxone had no effect in a second Na_V_1.7-null participant (male 2). The control participant shows higher perceived pain intensity, which is not enhanced by naloxone. For (B) and (C), the error bars represent standard error of the mean. Mean latencies before and after drug treatment were compared using repeated-measures two-way ANOVA followed by post hoc Sidak’s test. n = 9 animals for WT and n = 9 animals for KO^Adv^. See also Figure S6 and Table S1.

Is suppression of synaptic transmission by the opioid system required for analgesia? To test this in awake behaving animals, we selectively blocked opioid receptors in the peripheral, central, or both compartments of the nociceptor (Figure 8A). We measured withdrawal latencies to radiant heat stimuli as a readout. To test whether central opioid receptors are required, we injected naloxone (3 mM in 5 μL) by intrathecal injection into the lumbar spinal column. Centrally administered naloxone was sufficient to reverse thermal hyposensitivity in Na_V_1.7 KO^Adv^ mice by 76%. There was no effect of intrathecal vehicle injection (Figure 8B). Naloxone methiodide is a peripherally restricted naloxone analog that does not cross the blood-brain barrier and, hence, selectively blocks peripheral opioid receptors (Melo et al., 2018). Systemic administration of naloxone methiodide (2 mg/kg) had no effect on withdrawal latencies in WT or KO^Adv^ mice, indicating that peripheral opioid receptors are dispensable for analgesia linked to Na_V_1.7 loss of function (Figure 8C). In contrast, systemic injection of naloxone (2 mg/kg) caused a 71% reversal of analgesia in the KO^Adv^ group. Thus, central, but not peripheral, opioid receptors are essential for maintenance of analgesia in mice lacking Na_V_1.7.

We have shown before that naloxone infusion restored nociception in a single rare female Na_V_1.7-null human (Minett et al., 2015). Here we extended these findings to two male humans with compound heterozygous Na_V_1.7 loss-of-function mutations (Table S1; McDermott et al., 2019; Ramirez et al., 2014; Shaikh et al., 2018). We applied tonic radiant heat stimuli (25 s) to the forearm while participants rated the perceived intensity online using a visual analog scale (Figure 8D). In male 1, naloxone strongly enhanced pain sensation, mimicking the effect on the previously reported female participant (Minett et al., 2015). In male 2, there was no apparent effect of naloxone. In an age- and gender-matched control individual, pain ratings were consistently higher than in the Na_V_1.7-null individuals and not enhanced by naloxone. Thus, in 2 of the 3 humans with Na_V_1.7-null mutations we tested so far, blockade of opioid receptors enhances pain sensitivity, supporting a role of opioid signaling in driving analgesia associated with Na_V_1.7 loss of function.

## Discussion

Deletion of *SCN9A* encoding the peripheral sodium channel Na_V_1.7 in humans causes pain insensitivity and anosmia (Cox et al., 2006; Weiss et al., 2011). Peripheral neuron Na_V_1.7 KO mice are pain free in assays of mechanical, heat, and inflammatory pain and show decreased spinal cord wide-dynamic-range neuron firing to noxious stimuli (Minett et al., 2012; Nassar et al., 2004). The main driver of analgesia must thus be loss of Na_V_1.7 in sensory neurons rather than the CNS. Because Na_V_1.7 exhibits slow closed-state inactivation, the channel is less likely to inactivate during slow sub-threshold depolarizations. Na_V_1.7 therefore mediates a ramp current that is hypothesized to amplify noxious stimulus-induced generator potentials to trigger action potential firing at nociceptor peripheral terminals (Cummins et al., 1998). In patch-clamp studies of cultured mouse sensory neurons and human induced pluripotent stem cell (iPSC)-derived nociceptors under certain recording conditions, Na_V_1.7 deletion impairs action potential firing upon depolarization of the soma (McDermott et al., 2019; Shields et al., 2018). Recording of compound action potentials in KO^Adv^ mice also suggests some deficits in peripheral excitability (Hoffmann et al., 2018). Hence, the prevailing hypothesis and rationale for developing peripherally targeted inhibitors is that analgesia after Na_V_1.7 loss of function arises from reduced excitability of the nociceptor peripheral terminal.

Using *in vivo* imaging and electrophysiology, we directly tested whether loss of Na_V_1.7 causes peripheral silencing of nociceptors in live mice. To our surprise, nociceptor excitability at the level of the dorsal root ganglion (DRG) was largely unchanged, with normal levels of heat, polymodal and silent nociceptors, and normal calcium and spike response profiles. A hallmark of CIP associated with Na_V_1.7 loss of function is painless burns, but DRG responses to an unequivocally noxious heat stimulus (55°C) were unaffected by Na_V_1.7 deletion. Moreover, we observed intact peripheral sensitization of nociceptor activity following inflammatory mediator injection in Na_V_1.7 KO mice despite sensitization of behavioral responses to the same treatment being abolished. Nonetheless, we did find reduced numbers of cells responding to noxious mechanical stimuli, consistent with earlier *in vitro* recordings that found that ∼30% of Na_V_1.7-deficient putative nociceptors are electrically silenced in culture (Raouf et al., 2012). In the main, however, action potentials propagate as far as the soma in Na_V_1.7-deleted sensory neurons, indicating that the locus of analgesia is unlikely to lie at the peripheral terminal, explaining the failure of peripherally targeted inhibitors of Na_V_1.7 to relieve pain (Kingwell, 2019).

Studies of humans with loss-of-function mutations in Na_V_1.7 posit die-back of peripheral nerves as a significant mechanism of analgesia (Marchi et al., 2018; McDermott et al., 2019; Nilsen et al., 2009). In three human Na_V_1.7-null individuals, microneurographic recordings found evidence of Aδ but not C fibers, based on activity-dependent slowing profiles (McDermott et al., 2019). These subjects were not challenged with painful stimuli. Nociceptors encompass all classes of sensory fibers, including Aβ fibers, and are defined by their ability to respond to and encode noxious stimuli (Emery and Wood, 2019; Lawson et al., 2019). Preservation of neurogenic inflammation combined with a case report of a child with normal epidermal innervation and loss of pain are inconsistent with neuropathy as the principal mechanism of analgesia (Klein et al., 2013; McDermott et al., 2019). Na_V_1.7-null individuals also lose the channel from innocuous touch-sensing neurons that function normally and do not seem to die back (Zeisel et al., 2018).

Na_V_1.7 is expressed along the length of sensory neurons, including at the central terminal, where it associates with many proteins, including synaptotagmin-2 (Black et al., 2012; Kanellopoulos et al., 2018). Given normal peripheral excitability but reduced spinal cord neuron firing in Na_V_1.7 KOs, an alternative analgesic mechanism is that nociceptive input is lost through failure of synaptic transmission from nociceptors to CNS neurons. Indeed, application of Na_V_1.7 inhibitors to spinal cord slices reduces synaptic transmission from afferents (Alexandrou et al., 2016; Deuis et al., 2017). Using glutamate imaging and electrophysiology, we observed deficits in pain-related neurotransmitter release in the spinal cord of mice lacking Na_V_1.7. Although stimulus current thresholds for evoked glutamate release were generally in the C-fiber range for WT animals, unnaturally high stimulus intensities were often required to elicit any release in KOs, consistent with the increased sensory thresholds for reflexive withdrawal behavior seen in these animals. Because we directly activated dorsal roots of spinal cord slices, the glutamate imaging experiments preclude any contribution of impaired peripheral excitability to the observed synaptic deficits. Echoing this, direct stimulation of dorsal roots in one individual with CIP failed to elicit pain (Manfredi et al., 1981).

How does loss of Na_V_1.7 impair neurotransmitter release? Analgesia in mice and humans lacking Na_V_1.7 can be reversed substantially by opioid antagonists or opioid receptor deletion. This results from enhanced PENK production and opioid receptor signaling (Isensee et al., 2017; Minett et al., 2015; Pereira et al., 2018). Interestingly, regulation of *Penk* transcription and opioid receptor signaling has been linked to lowered sodium levels that may contribute to Na_V_1.7 loss-of-function analgesia (Isensee et al., 2017; Minett et al., 2015). Because opioids are known to potently suppress neurotransmitter release from spinal cord afferent terminals, we wondered whether the synaptic impairments we saw in Na_V_1.7 KOs are dependent on opioid receptors (Heinke et al., 2011; Yaksh et al., 1980). Peripheral excitability was unaffected by the opioid blocker naloxone in Na_V_1.7 KOs, consistent with previous findings using Na_V_1.7-deficient iPSC-derived human nociceptors (McDermott et al., 2019). The deficits in glutamate transmission and WDR input within the spinal cord were, however, reversed by naloxone. μ and δ opioid receptors are expressed at the central terminals in non-overlapping sets of nociceptors (Corder et al., 2017; Scherrer et al., 2009). This is consistent with our previous finding that combined genetic deletion or pharmacological blockade of μ and δ, but not κ, opioid receptors is required to reverse Na_V_1.7-null analgesia (Pereira et al., 2018). Interestingly, met-enkephalin levels are also increased in the dorsal horn of Na_V_1.7 KO mice (Minett et al., 2015).

Anosmia in Na_V_1.7 nulls is wholly explained by impaired synaptic transmission from first-order olfactory sensory neurons, although somatic excitability to odorant stimuli is normal (Weiss et al., 2011). We found that synaptic transmission from olfactory sensory neurons was not rescued by naloxone treatment. This is not surprising, given that olfactory sensory neurons do not express opioid receptors (Saraiva et al., 2015). Na_V_1.7 is the only sodium channel available in olfactory sensory neuron nerve terminals; thus, its loss completely blocks electrical activity presynaptically, consistent with abolition of transmitter release by TTX (Ahn et al., 2011; Weiss et al., 2011). In contrast, nociceptors express other sodium channels that can support synaptic transmission in the absence of Na_V_1.7 when the inhibitory effect of opioids is removed (Medvedeva et al., 2009; Vysokov et al., 2019).

Opioid action at the central terminal is causally involved in pain insensitivity because central administration of naloxone was sufficient to substantially reverse analgesia but systemic administration of a peripherally restricted opioid antagonist was not. In humans, naloxone infusion enhanced sensitivity to nociceptive stimuli in 2 of the 3 Na_V_1.7-null individuals tested so far. Why did naloxone have no effect on heat sensitivity in male 2? This participant is hyposensitive to warmth and cooling. Interestingly, during adolescence he developed the ability to avoid injury by detecting a “tingling” sensation when exposed to noxious thermal and mechanical stimuli (Ramirez et al., 2014). In an experimental setting, the reported intensity of tingling encoded the strength of the stimulus for temperatures above 42°C but was never perceived as unpleasant. These unusual phenotypic characteristics could affect his perception of the tonic heat stimulus used here, which he rated as zero throughout. Importantly, numerous early case reports in the clinical literature attest to the dependence of CIP-like phenotypes on the opioid system, whilst sodium channel blockers show synergistic analgesia when paired with opioid drugs (Dehen et al., 1977; Kolesnikov et al., 2000; Vetter et al., 2017). In one particularly elegant experiment, intrathecal injection of cerebrospinal fluid (CSF) from an unmapped individual with CIP reduced heat nociception in rats. Because this effect was blocked by naloxone, CSF opioids acting centrally are sufficient to recapitulate some CIP-associated analgesia in animals (Fabbri et al., 1984).

What are the implications of a central, opioid-dependent mechanism of analgesia for pain therapies targeting Na_V_1.7? According to recent single-cell RNA sequencing studies, Na_V_1.7 is expressed widely in all sensory neuron subtypes, apart from proprioceptors (Zeisel et al., 2018). However, mice and humans lacking Na_V_1.7 are insensitive only to noxious stimuli. If Na_V_1.7 deletion results in electrical silencing of sensory neurons, then why is touch sensation not lost? Single-cell RNA sequencing data show that opioid receptors are not expressed by neurons expressing markers for low-threshold mechanoreceptors (Zeisel et al., 2018). We suggest that only in cells expressing opioid receptors can Na_V_1.7 deletion drive impaired synaptic transmission by enhancing opioid receptor signaling. Importantly, deletion of the transcription factor NFAT5 leads to elevated *Penk* mRNA transcripts with no analgesia (Pereira et al., 2018). Enhanced opioid receptor signaling is therefore a crucial component of analgesia in Na_V_1.7 KO mice. This is consistent with recent observations that peptide antagonists of Na_V_1.7 that elicit analgesia in mice can be inhibited by naloxone (Chen et al., 2018; Mueller et al., 2019). Profound analgesic synergy between Na_V_1.7 blockers and low-dose opioids has also been reported (Deuis et al., 2017; Emery et al., 2016a). Na_V_1.7 blockade sensitizes opioid signaling only in nociceptors, reducing the effective concentration of opioids required to inhibit synaptic transmission from terminals. Close to 100% channel blockade may be required to drive the changes in opioid function responsible for analgesia (Minett et al., 2015). For intractable chronic pain, gene therapy strategies that mimic genetic loss of function will likely be required (Moreno et al., 2021).

These data support a mechanism where pain insensitivity of mice and humans lacking Na_V_1.7 principally involves opioid signaling (Figure S6). In contrast, anosmia in Na_v_1.7 null mutants is opioid independent. Diminished peripheral excitability and die-back may also contribute to analgesia, but opioid-mediated suppression of neurotransmitter release plays a major role. Interestingly, a combination of highly specific Na_v_1.7 antagonists and opioids causes potent analgesia in mice, whereas each component is inactive alone (Deuis et al., 2017; Mueller et al., 2019). Endogenous opioids inhibit synaptic communication between the central terminals of peripheral nociceptors and post-synaptic neurons in the spinal cord, resulting in diminished nociceptive input to the CNS. Other mechanisms may contribute to Nav1.7 analgesia to a lesser extent. For example, dorsal horn neuron-intrinsic excitability is also reduced in Na_V_1.7-null mice through lack of transfer of the channel from primary afferent neurons to dorsal horn neurons (Alles et al., 2020). In addition, studies of hypothalamic neurons have shown the ability of Na_v_1.7 to integrate small depolarizations over long time periods to produce action potentials, a potentially significant mechanism still unexplored in nociceptive neurons (Branco et al., 2016).

We have demonstrated that nociceptor activity at the level of the DRG is largely unaffected by Na_V_1.7 deletion despite behavioral analgesia. The critical locus of analgesia in Na_V_1.7 nulls is therefore the central terminal and not, as thought previously, the periphery. Our findings consequently provide a biological explanation for the failure of peripherally targeted Na_V_1.7 inhibitors to cause analgesia and point to central terminal Na_V_1.7 and associated opioid signaling pathways as alternative therapeutic targets for pain relief.

## STAR★Methods

### Key resources table

**Table:** 

REAGENT or RESOURCE	SOURCE	IDENTIFIER
**Bacterial and virus strains**		

pAAV.CAG.GCaMP6f.WPRE.SV40	Chen et al., 2013	Addgene AAV1; 100836-AAV1
pAAV.hSyn.iGluSnFr.WPRE.SV40	Marvin et al., 2013	Addgene AAV9; 98929-AAV9

**Chemicals, peptides, and recombinant proteins**		

PF 05089771	Tocris	5931; CAS: 1430806-04-4
Prostaglandin E2	Merck (Sigma)	P0409; CAS: 363-24-6
Naloxone hydrochloride dihydrate	Merck (Sigma)	N7758; CAS: 51481-60-8
Naloxone Methiodide	Merck (Sigma)	N129; CAS: 93302-47-7

**Deposited data**		

*In vivo* imaging and electrophysiology – Source Data	Mendeley Data	https://doi.org/10.17632/5cw99c3w8p.1

**Experimental models: Organisms/strains**		

Mouse: Pirt-GCaMP3	Kim et al., 2014	N/A
Mouse: Na_V_1.7 flox	Nassar et al., 2004	N/A
Mouse: Advillin Cre	Zhou et al., 2010	N/A
Mouse: Wnt1 Cre	Danielian et al., 1998	N.A
Mouse: OMP Cre Na_V_1.7 KO	Weiss et al., 2011	N/A

**Oligonucleotides**		

Primers for mouse genotyping	See Table S2	N/A

**Software and algorithms**		

Fiji	ImageJ	https://fiji.sc/
R studio	R	https://rstudio.com/
LasX	Leica	https://www.leica-microsystems.com/
PrairieView	Bruker	https://www.bruker.com/en.html
pCLAMP	Molecular Devices	https://www.moleculardevices.com/
Prism	Graphpad	https://www.graphpad.com/

### Resource availability

#### Lead contact

Further information and requests for resources and reagents should be directed to and will be fulfilled by the lead contact, John N. Wood (j.wood@ucl.ac.uk).

#### Materials availability

This study did not generate any new unique reagents.

#### Data and code availability

Data are available from the lead contact on reasonable request. Source data for *in vivo* imaging and electrophysiology are deposited with Mendeley Data at https://doi.org/10.17632/5cw99c3w8p.1.

### Experimental model and subject details

#### Animals

All animal procedures carried out at University College London were approved by University College London ethical review committees and conformed to UK Home Office regulations. All animal procedures carried out at the University of Saarland were approved by the Institutional Animal Care and Use Committee of the University of Saarland (UdS) School of Medicine and were in accordance with the laws for animal experiments issued by the German Government.

The following mouse lines were used in this study: Advillin-Cre Na_V_1.7 KO, Wnt1-Cre Na_V_1.7 KO, Advillin-Cre Na_V_1.7 KO Pirt-GCaMP3, Wnt1-Cre Na_V_1.7 KO Pirt-GCaMP3 and OMP-Cre Na_V_1.7 KO. Breeding strategies were as previously described (Minett et al., 2012; Weiss et al., 2011). Peripheral Na_V_1.7 knockout mice expressing GCaMP3 were generated by crossing knockout animals with mice homozygous for floxed Na_V_1.7 and homozygous for Pirt-GCaMP3 (Kim et al., 2014). Mice were housed on a 12:12 hour light-dark cycle with food and water available *ad libitum*. For genotyping, genomic DNA was isolated from ear tissue or tail clip biopsy for PCR. Genotyping primers are summarized in Table S2. Both male and female animals were used for all experiments. Adult or late juvenile stage mice were used for all experiments, except for glutamate imaging studies which used tissue from animals pre-weaning, and specific age ranges for each experiment can be found in the Method details. The number of animals used to generate each dataset is described in individual figure legends.

#### Human subjects

We tested two male Na_V_1.7 null participants (aged 32 and early 30 s) and one age and gender matched (33) healthy control. All participants gave written informed consent. The study was approved by the UCL Research Ethics Committee. Both males are compound heterozygous nulls and have been previously described (McDermott et al., 2019; Ramirez et al., 2014). Mutations are summarized in Table S2.

### Method details

#### Viral injections

Neonatal pups (P1-P3) were injected with 5 μl AAV1-CAG-GCaMP6f or AAV9-Synapsin-iGluSnFR via the intraperitoneal route using a Hamilton syringe connected to a 30G needle cannula. Care was taken to minimize exposure to foreign scents to ensure re-acceptance of pups by parents upon return to breeding cage.

#### *In Vivo* Calcium Imaging

##### Acquisition

Mice expressing GCaMP3 or GCaMP6f (8 to 14 weeks, male and female) were anesthetized using ketamine (120 mg/kg) and medetomidine (1.2 mg/kg). Depth of anesthesia was confirmed by pedal reflex and breathing rate. Animals were maintained at a constant body temperature of 37°C using a heated mat (VetTech). Lateral laminectomy was performed at spinal level L3-5. In brief, the skin was incised longitudinally, and the paravertebral muscles were cut to expose the vertebral column. Transverse and superior articular processes of the vertebra were removed using microdissection scissors and OmniDrill 35 (WPI). To obtain a clear image of the sensory neuron cell bodies in the ipsilateral dorsal root ganglion (DRG), the dura mater and the arachnoid membranes were carefully opened using microdissection forceps. The animal was mounted onto a custom-made clamp attached to the vertebral column (L1), rostral to the laminectomy. The trunk of the animal was slightly elevated to minimize interference caused by respiration. Artificial cerebrospinal fluid [containing 120 mM NaCl, 3 mM KCl, 1.1 mM CaCl_2_, 10 mM glucose, 0.6 mM NaH_2_PO_4_, 0.8 mM MgSO_4_, 18 mM NaHCO_3_ (pH 7.4) with NaOH] was perfused over the exposed DRG during the procedure to maintain tissue integrity, or the DRG was isolated by coating with silicone elastomer.

Images were acquired using a Leica SP8 confocal microscope (Leica). GCaMP3 or GCaMP6f was excited using a 488 nm laser (1%–15% laser power). Images were acquired at 800Hz, with bidirectional laser scan. Typically, the pinhole was kept at 1 A.U, but in some experiments was increased to 1.5 A.U. to enhance brightness. Images magnification was between 0.75x and 3x optical zoom on a 10x air objective, depending on DRG anatomy. Noxious and innocuous stimuli were applied to the left hindpaw, ipsilateral to the exposed DRG. For thermal stimuli, the paw was immersed with ice-water (0°C) or water heated to 37°C or 55°C using a Pasteur pipette. For mechanical stimuli, we used noxious pinch with serrated forceps. PGE2 (500 μM in saline) was applied to the paw by intraplantar injection. Naloxone (2 mg/kg in saline) was delivered by subcutaneous injection into the scruff of the neck.

##### Analysis

Image stacks were registered to the first frame in the series using the FIJI plugin TurboReg (accurate rigid body transformation) to correct for XY drift. Stacks that showed excessive Z movement were excluded from analysis. Regions of interest (ROI) were manually drawn around apparently responding cells using the free hand tool in FIJI. Mean pixel intensity over time for each ROI was extracted and analyzed. The time series of mean pixel intensity for each ROI was smoothened by a four time point moving average to remove high-frequency noise. Next, we calculated the derivative of the mean pixel intensity. We calculated a mean baseline derivative for the 10 s preceding stimulus application. Neurons were classed as responders if, within 30 s of stimulus application, the maximum derivative was greater than the baseline derivative plus five standard deviations – that is, a Z-score of at least 5. We then calculated the ΔF/F_0_ value for each response to obtain a normalized measure of change in fluorescence. Neurons which showed a ΔF/F_0_ less than 0.25 were then discarded. Each trace was then manually screened as a further precaution against false positives. The remaining neurons that made up the responding population were then used for statistical analysis.

#### Behavioral Testing

All animal experiments were performed in accordance with Home Office Regulations. Observers were blinded to treatment and/or genotype. Animals were acclimatized to handling by the investigator and every effort was made to minimize stress during the testing. Both male and female animals were used.

##### Randall Selitto

The threshold for mechanonociception was assessed using the Randall Selitto test (Randall and Selitto, 1957). Animals were restrained in a clear plastic tube. A 3 mm^2^ blunt probe was applied to the tail of the animal with increasing pressure until the mouse exhibited a nocifensive response, such as tail withdrawal. The pressure required to elicit nocifensive behavior was averaged across three trials. The cut-off was 500 g.

##### Von Frey

Punctate mechanical sensitivity was measured using the up-down method of Chaplan to obtain a 50% withdrawal threshold (Chaplan et al., 1994). Mice were habituated for one hour in darkened enclosures with a wire mesh floor. A 0.4 g Von Frey filament was applied to the plantar surface of the paw for 3 s. A positive response resulted in application of a filament of lesser strength on the following trial, and no response in application of a stronger filament. To calculate the 50% withdrawal threshold, five responses surrounding the 50% threshold were obtained after the first change in response. The pattern of responses was used to calculate the 50% threshold = (10[χ+κδ])/10,000), where χ is the log of the final von Frey filament used, κ = tabular value for the pattern of responses and δ the mean difference between filaments used in log units. The log of the 50% threshold was used to calculate summary and test statistics, in accordance with Weber’s Law.

##### Hargreaves’ Test:

Spinal reflex responses to noxious heat stimulation were assessed using the Hargreaves’ test (Hargreaves et al., 1988). Mice were habituated for an hour in plexiglass enclosures with a glass base. Before testing, the enclosures were cleaned of faeces and urine. Radiant heat was then locally applied to the plantar surface of the hindpaw until the animal exhibited a nocifensive withdrawal response. Average latencies were obtained from three trials per animal, with inter-trial interval of 15 mins. Cut-off time was 30 s. The effect of intraplanar PGE2 (500 μM) on heat sensitivity was assessed using the Hargreaves’ test. A baseline withdrawal latency was obtained and then measured again 10 minutes following PGE2 treatment. The effect of subcutaneous opioid blockers (2 mg/kg for 20 minutes) or intrathecal naloxone (3 mM in 5 μl) was also assessed using this assay, 20 minutes following drug treatment. For intrathecal injections, mice were anesthetized using 2%–3% isofluorane and drugs delivered via a 30G needle cannula attached to a Hamilton syringe.

##### Cold Plantar

Spinal reflex responses to cooling were assessed using the Cold Plantar test (Brenner et al., 2012). Mice were placed in Plexiglass enclosures with glass flooring and acclimatized for one hour. Before testing, faeces and urine were removed and the animal was left to settle. Dry ice was compacted into a blunt 2 mL syringe and applied to the glass surface just below the hindpaw. The time to withdrawal was measured. Cut off was 30 s. Testing was repeated 3 times, and averaged, with a waiting period of 15min between stimulations.

#### Electrophysiology

##### In vitro electrophysiology

Mice were killed by inhalation of a rising CO_2_ concentration followed by cervical dislocation to confirm death. Dorsal root ganglia were dissected and then digested in an enzyme mix for 45 minutes before mechanical trituration. Neurons were re-suspended in DMEM supplemented with nerve growth factor and plated onto 12 mm glass coverslips coated with poly-l-lysine/laminin.

Patch pipettes (tip resistance of 3-5 MΩ) were filled with intracellular solution containing: 140 mM CsF, 1 mM EGTA, 5 mM NaCl and 10 mM HEPES. To isolate macroscopic sodium currents, neurons were continuously perfused with room temperature extracellular solution containing: 35 mM NaCl, 75 mM Choline-Cl, 30 mM TEA-Cl, 4 mM KCl, 1.8 mM CaCl_2_, 1 mM MgCl_2_, 10 mM HEPES, 5 mM Glucose and 0.1 mM CdCl_2_. Whole-cell recordings were obtained using an Axopatch 200B amplifier, filtered at 10 kHz and digitized at 50 kHz via a Digidata 1322A (Axon Instruments). Medium diameter neurons from WT and Na_V_1.7 KO mice were voltage-clamped at −70 mV. Series resistance compensation was at least 60%. To measure the voltage-dependence of sodium channel activation, the holding command was dropped to −120 mV to de-inactivate all sodium channels and then a step-protocol from −80 to 20 mV was applied, in increments of 5 mV, to activate sodium channels. To determine the contribution of Na_V_1.7 to the total sodium current, the Na_V_1.7 blocker PF 05089771 was applied for 5 minutes at 100 μM. As PF 05089771 is a state-dependent blocker that binds only to the inactivated state of the channel, the holding command was increased to −40 mV to inactivate sodium channels for the duration of drug application.

##### Ex vivo spinal cord slice electrophysiology

Spinal cord preparations were obtained from male or female mice, between 30 and 60 days old, from either wild-type C75Bl/6 (WT) or conditional Na_V_1.7 knockout (Na_V_1.7 KO). Animals were anesthetized via intraperitoneal injection of a ketamine/xylaxine mix (80 mg/kg and 10 mg/kg respectively) and decapitated. The spinal cord was dissected in ice cold aCSF containing: 113 mM NaCl, 3 mM KCl, 25 mM NaHCO3, 1 mM NaH2PO4, 2 mM CaCl2, 2 mM MgCl2, and 11 mM D-glucose. Once dissected free from the vertebral column, the spinal cord was carefully cleaned from connective tissues and dorsal roots were cut at approximately 2 mmm length. The spinal cord was then glued to an agar block and glued to the slicing chamber of a HM 650V vibratome (Microm, ThermoFisher Scientific, UK). The slicing solution contained: 130 mM K-gluconate, 15 mM KCl, 0.05 mM EGTA, 20 mM HEPES, 25 mM D-glucose, 3 mM kynurenic acid, 2 mM Na-Pyruvate, 3 mM Myo-Inositol, 1 mM Na-L-Ascorbate, and pH 7.4 with NaOH_4_. Slices were incubated for 40 minutes at 35 degrees and then allowed to equilibrate at room temperature for further 30 minutes before starting the recordings.

Voltage clamp recordings were performed using either a Molecular Devices Multiclamp 700B (Scientifica, UK) or an ELC-03X amplifier (NPI electronics, Germany). Signals were filtered at 5KHz, acquired at 50 KHz using a Molecular Devices 1440A A/D converter (Scientifica, UK) and recorded using Clampex 10 software (Molecular Devices, Scientifica, UK). Electrodes were pulled with a Flaming-Brown puller (P1000, Sutter Instruments, USA) from borosilicate thick glass (GC150F, Harvard Apparatus, UK). The resistance of the electrodes, following fire polishing of the tip, ranged between 3 and 5 MΩ. Bridge balance was applied to all recordings. Intracellular solution contained 125 mM K-gluconate, 6 mM KCl, 10 mM HEPES, 0.1 mM EGTA, 2 mM Mg-ATP, pH 7.3 with KOH, and osmolarity of 290–310 mOsm. Cells were targeted for patching in the inner and outer Lamina II and visualized through an Eclipse E600FN Nikon microscope (Nikon, Japan) equipped with infrared differential interference contrast (IR-DIC) connected to a digital camera (Nikon, DS-Qi1Mc). Cells were voltage-clamped at −70 mV and spontaneous excitatory post synaptic currents (sEPSCs) recorded. sEPSCs were automatically detected using ClampFit in a 5 s window for each cell.

##### Ex vivo olfactory bulb electrophysiology

Acute MOB slices were prepared from 4 - 11 week old mice (male and female) anesthetized with CO_2_ before decapitation. OBs were rapidly dissected in ice-cold oxygenated (95% O_2_, 5% CO_2_) solution containing the following (in mM): 83 NaCl, 26.2 NaHCO3, 1 NaH2PO4, 2.5 KCl, 3.3 MgCl_2_, 0.5 CaCl_2_, 70 sucrose, pH 7.3 (osmolarity, 300 mOsm/l). The tissue was mounted on a vibratome (VT1000S; Leica Microsystems, Nussloch, Germany) and horizontal MOB slices (275 μm thick) were cut in the same solution. Slices were stored at 30 - 35°C for 15 - 20 min in standard extracellular solution and afterward at room temperature until use. The extracellular solution contained the following (in mM): 125 NaCl, 25 NaHCO_3_, 2.5 KCl, 1.25 NaH_2_PO_4_, 1 MgCl_2_, 2 CaCl_2_ and 10 glucose (continuously bubbled with 95% O_2_, 5% CO_2_). Tissue slices were placed in the recording chamber and superfused at a rate of ∼2 ml/min (gravity flow) with extracellular solution bubbled with carbogen (95% O_2_, 5% CO_2_). Cells were visualized in intact tissue slices with a 40x water immersion objective lens (Olympus) using infrared-optimized differential interference contrast optics and fluorescent illumination and a GFP filter set attached to the microscope to elucidate the morphology of lucifer yellow-filled mitral and tufted cells (BX50WI, Olympus).

Slice recordings were carried out at room temperature using an EPC-9 automated patch-clamp amplifier (HEKA Elektronik, Lambrecht, Germany) and Pulse 8.11 software as described previously (Weiss et al., 2011). Patch pipettes were pulled from borosilicate glass tubing (World Precision Instruments, Germany). The signals were filtered using an eight-pole Bessel filter built into the EPC-9 amplifier and digitized at a frequency ≥ filter cut-off frequency (VR-10B, Instrutech Corp.). The sampling rate during all recordings was 10 kHz. Recording pipettes had resistances of 3 - 6 MΩ. Cells were voltage-clamped in the whole-cell patch-clamp mode. M/T cells had an ellipsoid-shaped cell body with a diameter of > 10 μm, were located in the mitral cell layer or in the external plexiform layer. We did not discriminate between mitral cells and tufted cells within the group of M/T cells. M/T cells were filled with lucifer yellow during the recording and were afterward visually inspected using fluorescent illumination.

The intracellular solution contained (in mM): 140 CsCl, 1 EGTA, 10 HEPES, 2 ATP Na-salt, 1 GTP Mg-salt, 5 QX-314 (a lidocaine derivative; Sigma-Aldrich, Taufkirchen, Germany), 0.1 lucifer yellow, 0.4 neurobiotin (Vector Laboratories, Burlingame, CA, USA); pH 7.1; osmolarity 290 mosm). The theoretical liquid junction potential between intracellular and extracellular compartments was calculated to be 4.1 mV and was not corrected.

After establishing a whole-cell recording, cells were voltage clamped to −60 mV. We waited for at least 2 min before data acquisition began to allow for equilibration of intracellular solution into the dendrites. Electrical stimulation of the olfactory nerve layer was applied via a glass electrode filled with extracellular solution and connected to an Isolated Pulse Stimulator Model 2100 (A-M Systems Instruments, USA). Electrodes were visually positioned in close proximity to the corresponding glomerulus of the recording site and stimulus duration and intensity was 1 ms and 100 V, respectively. Extracellular solution containing the opioid receptor antagonist naloxone (300 μM, Sigma Aldrich, Germany) was perfused to the MOB slice for at least 10 minutes.

All electrophysiological data were analyzed using Igor Pro software (WaveMetrics) and Excel (Microsoft). For pharmacological experiments, amplitudes of evoked EPSCs were assessed. The Student’s t test was used to measure the significance of difference between two distributions. Data are expressed as means ± SEM.

##### In vivo electrophysiology

Electrophysiological recordings were performed by a blinded experimenter. Mice were anaesthetized with isofluorane (4%; 0.5 l/min N_2_O and 1.5 l/min O_2_) before being secured in a stereotaxic frame. Depth of anesthesia was reduced and maintained at 1.5% isoflurane during the experiment. For DRG recordings, lateral laminectomy was performed to expose the L4 DRG, as described above for *in vivo* imaging. For spinal cord, a laminectomy was performed to expose L3–L5 segments of the spinal cord and extracellular recordings were made from WDR neurons in the deep dorsal horn (lamina III–V, 200–600 μm). Multi-unit extracellular recordings were made from DRG neurons or WDR neurons using parylene-coated tungsten electrodes (A-M Systems). Mechanical and thermal stimuli were applied to the peripheral receptive field of hindpaw glabrous skin ipsilateral to the exposed DRG. Natural stimuli (dynamic brush, von Frey hairs 0.16–26 g, noxious prod 100 and 150 g/cm^2^ mechanical stimulation, thermal water jet 35–55°C and iced water) were applied in ascending order of intensity to receptive fields for 10 s and the total number of evoked spikes recorded. Ethyl chloride was applied for 1 s as a noxious cold stimulus and the total number of evoked spikes in 10 s was quantified. Evoked activity of neurons was visualized on an oscilloscope and discriminated on spike amplitude and waveform basis using a CED 1401 interface coupled to Spike2 software (Cambridge Electronic Design) to record waveform templates and carry out principal component analysis. For naloxone experiments, 2 mg/kg naloxone in saline was injected subcutaneously into the scruff of the neck, and the stimulation protocol repeated again 20 minutes after naloxone injection.

#### Glutamate Imaging

##### In vitro characterization

Dorsal root ganglia neurons were dissociated and cultured onto glass coverslips as described above. Neurons were treated with iGluSnFR AAV particles diluted in culture media at dilutions varying from 1/100 to 1/4000. After 2-5 days treatments, neurons expressed the virus at all tested dilutions. Coverslips were transferred to an imaging chamber and perfused with extracellular solution containing: 140 mM NaCl, 4 mM KCl, 1.8 mM CaCl2, 1 mM MgCl2, 10 mM HEPES and 5 mM glucose, with pH 7.4. Images were acquired using a Leica SP8 confocal microscope with 20x immersion objective and iGluSnFR was excited using a 488 nm laser (1%–5% laser power). Glutamate was applied at various concentrations in the bathing solution resulting in fluorescence increases localized to the plasma membrane. For analysis, ring-shaped regions of interest were drawn around the membrane and mean pixel intensity extracted and converted to ΔF/F_0_. Three-parameter dose-response curves were fit using GraphPad prism with a standard Hill Slope of 1.

##### Two-photon imaging

Lumbar spinal cord slices were prepared for glutamate imaging from P9-P21 mice virally expressing iGluSnFr in sensory afferents (Marvin et al., 2013). Mice were culled by intraperitoneal injection of a ketamine (60mg/kg) and xylazine (12mg/kg) cocktail followed by decapitation and exsanguination. Spinal cords were dissected in an ice-cold oxygenated (5% CO_2_ / 95% oxygen) dissection solution containing: 215 mM sucrose, 3 mM K-gluconate, 1.25 mM NaH_2_PO_4_, 26 mM NaHCO_3_, 4 mM MgSO_4_-7H_2_O, 10 mM d-glucose, 1 mM kynurenic acid and 1 mM CaCl_2_. Spinal cords were embedded in low-melting point agarose (2%–3%) in ASCF and then sectioned using a vibrating microtome into 500 um thick transverse slices in oxygenated ACSF. Slices were incubated for at least an hour in oxygenated ACSF at 37°C. The ACSF contained: 111 mM NaCl, 3.085 mM KCl, 10.99 mM d-glucose, 25 mM NaHCO_3_, 1.26 mM MgSO_4_-7H_2_O, 2.52 mM CaCl_2_, and 1.1. mM KH_2_PO_4_.

Slices with dorsal roots attached were transferred to the recording chamber and pinned using a harp. Using a 10x air objective on a Brüker 2P microscope, the dorsal roots were visualized and approached with the suction electrode. Roots were gently suctioned into the suction electrode, allowing a tight seal to form. An Isoflex (Molecular Devices) stimulus isolator was used to deliver negative current pulses of varying amplitudes. The stimulus isolator was triggered directly from the imaging software (Prairie View) and stimulation was timelocked to image acquisition. The temporal profile of the output waveform was controlled from the imaging software, with pulse duration of 400 μs. To ensure accurate time-locking, the output from the stimulus isolator was also recorded by the imaging software.

Images were acquired using a 2-photon microscope (Bruker) with a 20x high NA water immersion objective. iGluSnFr was excited using a 920 nm laser line (Insight DS, Spectra-Physics) and 525/70 nm emission acquired by a GaAsP PMT (Hamamatsu) with gain set to maximum. The location of layer II of the dorsal horn was estimated using physical and fluorescent landmarks and a 250 × 125 pixel field of view drawn, equivalent to 195 × 98 μm. Images of glutamate release in response to different single pulse stimulus intensities were acquired at 10 Hz. Throughout the experiments slices were perfused with oxygenated room temperature ACSF and, in some experiments, the bathing solution contained naloxone (100 uM), which was applied for at least 20 minutes.

##### Analysis

Image stacks were analyzed in Fiji. Trials were concatenated and then registered to the first image in the stack (accurate rigid body transformation). Regions of interest were manually identified by a blinded experimenter and the mean pixel intensity over time per ROI extracted. Signals were converted to ΔF/F0. Glutamate release events time-locked to the stimulus were z-scored and considered significant if z > 4. This dataset was then used for subsequent statistical analysis.

#### Immunohistochemistry

One day prior to the experiment, mice were distributed onto individual homecages. Naloxone (cat# N7758, Sigma Aldrich) was dissolved in phosphate buffered saline (PBS) pH 7.4 and systemically applied by intraperitoneal injection of 2 mg/kg bodyweight (Pereira et al., 2018), while negative controls received vehicle (PBS) alone. Tested mice (n = 3 each genotype) were Nav1.7^control^ [(flox/–)(OMP/–)(Cre/–)] and Nav1.7 KO^OMP^ [(fx/fx) (OMP/–)(Cre/–)] mice. For each mouse, three consecutive intraperitoneal injections with 30 min time intervals were performed. After each injection, mice were returned to their odour-rich home cages. At 24 hours after the last injection, mice were anesthetized, subjected to transcardial perfusion, followed by tissue preparation for tyrosine hydroxylase immunohistochemistry.

Mouse tissue preparation followed previously described methods (Weiss et al., 2011; Bolz et al., 2017). Adult mice (7-9 weeks-of-age) were anaesthetized using a mixture of 165 mg/kg body weight ketamine (Pharmacia GmbH, Berlin) and 11 mg/kg body weight xylazine (Bayer Health Care, Leverkusen), and were transcardially perfused with phosphate-buffered saline (PBS) pH 7.4, followed by 4% (w/v) paraformaldehyde in PBS. Olfactory bulbs (OBs) were dissected, incubated for 2 h in fixative and for overnight in 30% sucrose in PBS at 4°C, embedded in O.C.T. (Tissue-Tek), and snap-frozen in a dry ice/2-methylbutane bath. Frozen tissue sections (18 μm) were collected on a cryostat (Microm HM525, Walldorf, Germany) and stored at −80°C until subjected to immunohistochemistry. For tyrosine hydroxylase (TH) immunostaining, tissue sections were rinsed in PBS, treated for 1 h with blocking buffer containing 0.3% Triton X-100 and 4% normal horse serum (NHS, Vector Laboratories) prepared in PBS, followed by incubation in TH primary antibody (mouse monoclonal, cat# 22941, RRID:AB_572268, ImmunoStar, Hudson, WI, USA) diluted 1:2000 in blocking solution. Tissue sections were washed three times 10 min in PBS and incubated in Alexa Fluor 488 conjugated goat-anti-mouse secondary antibody (1:1000, Thermo Fisher Scientific cat# A-11029, RRID:AB_2534088) for 1 h in the dark. Tissue sections were rinsed in PBS, the nuclei counterstained with Hoechst 33342 (1:10,000 in PBS, Invitrogen) for 10 min, rinsed again in PBS, and coverslipped using fluorescence mounting medium (DAKO). All procedures were conducted at room temperature with the exception of tissue incubation in primary antibody solution at 4°C.

Confocal fluorescence images were acquired on a Zeiss LSM 880 confocal microscope containing a 32-channel GaAsP-PMT and 2-channel PMT QUASAR detector. Each image shown in Figure 6C refers to a single 2.5 μm thick optical section. Images were assembled and minimally adjusted in brightness using Adobe PhotoShop Elements 10.

#### Human Sensory Testing

Perception of tonic radiant heat was assessed at baseline and during intravenous administration of saline or naloxone (12 mg), in a randomized order. The perception of long-lasting, tonic nociceptive stimuli is generally considered to be less confounded by attention than transient noxious stimuli, involving rapid, attentional shifts that can confound perceptual measures (Legrain et al., 2011). Psychophysical assessment was carried out by an experimenter blind to the pharmacological condition. Tonic radiant heat was generated by a CO_2_ laser, whose power is regulated using a feedback control based on an online measurement of skin temperature at the site of stimulation (Laser Stimulation Device, SIFEC, Belgium). The CO_2_ laser selectively stimulates both A-delta and C fibers. On each trial, tonic radiant heat was delivered to the forearm for 25 s and kept constant at either 45 or 48 °C (Churyukanov et al., 2012). Participants were asked to rate the intensity of the thermal sensation on a visual analog scale throughout the trial (0 = no sensation, 100 = worst pain imaginable). Three trials per stimulus temperature were given on each session (baseline, saline and naloxone) in a randomized order.

### Quantification and statistical analysis

For *in vivo* imaging experiments, n refers to the number of cells responding to any stimulus. For electrophysiology experiments, n refers to the number of recorded cells. For glutamate imaging experiments, n refers to the number of regions of interest. For all imaging and physiology data, the number of animals used is indicated in the legend. For behavioral experiments, n refers to the number of animals.

Datasets are presented using appropriate summary statistics as indicated in the legend. Error bars denote mean ± 95% confidence interval or mean ± SEM, as indicated in the legend. The 95% confidence interval around proportions was estimated using the Wilson-Brown method. Tests of statistical comparison for each dataset are described in detail in figure legends. For grouped data, we made the appropriate correction for multiple comparisons. We set an α-value of p = 0.05 for significance testing and report all p values resulting from planned hypothesis testing.

No sample size calculation was performed, however our samples are similar to those used in the field.

## Supplemental information

Document S1. Figures S1–S6 and Tables S1 and S2

Document S2. Article plus supplemental information
