# Supplementary material for: A central mechanism of analgesia in mice and humans lacking the sodium channel NaV1.7
Source: Neuron. 2021 May 5;109(9):1497–1512.e6. doi: 10.1016/j.neuron.2021.03.012 (PMC8110947; doi:10.1016/j.neuron.2021.03.012)
Supplement: Document S1. Figures S1–S6 and Tables S1 and S2 [file mmc1.pdf]

Neuron, Volume *109*

Supplemental information

A central mechanism of analgesia in mice and humans lacking the sodium channel $Na_V1.7$

Donald Iain MacDonald, Shafaq Sikandar, Jan Weiss, Martina Pyrski, Ana P. Luiz, Queensta Millet, Edward C. Emery, Flavia Mancini, Gian D. Iannetti, Sascha R.A. Alles, Manuel Arcangeletti, Jing Zhao, James J. Cox, Robert M. Brownstone, Frank Zufall, and John N. Wood

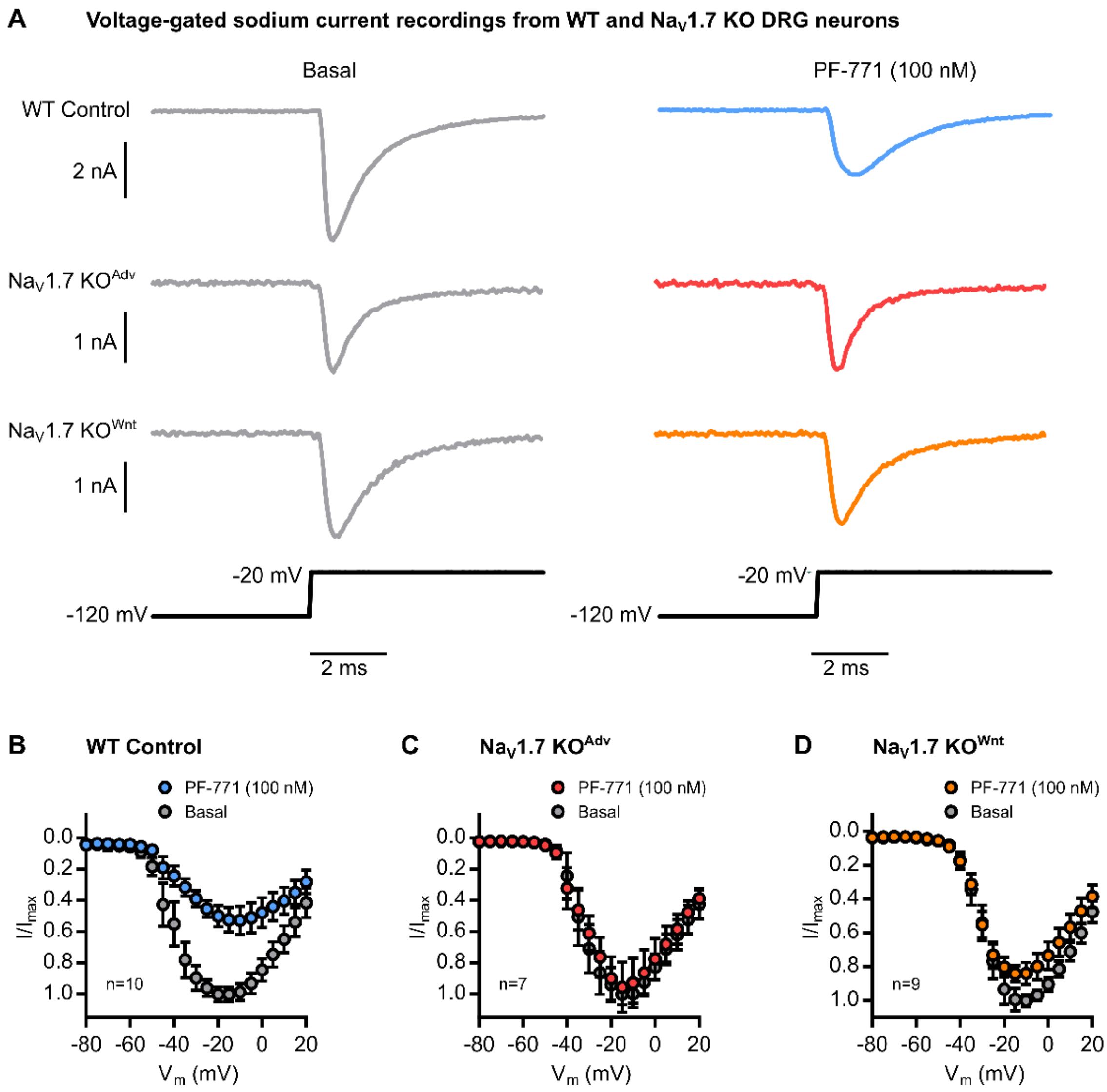
A
Voltage-gated sodium current recordings from WT and NaV1.7 KO DRG neurons
Basal
PF-771 (100 nM)
WT Control
2 nA
NaV1.7 KOAdv
1 nA
NaV1.7 KOWnt
1 nA
-20 mV
-120 mV
2 ms
B
WT Control
PF-771 (100 nM)
Basal
n=10
I/Imax
Vm (mV)
C
NaV1.7 KOAdv
n=7
D
NaV1.7 KOWnt
n=9

Figure S1 (*related to Figure 1*). Conditional deletion of *SCN9A* in sensory neurons abolishes voltage-gated sodium channel currents attributed to Na_V1.7.

(A) Example current traces showing voltage-gated sodium channel currents activated by a 100 mV voltage-step from -120 mV to 20 mV recorded from WT and Na_V1.7 KO DRG neurons (grey). Application of the Na_V1.7-specific antagonist PF-05089771 (PF-771, 100 nM for 5 minutes) caused a marked reduction in sodium currents in WT neurons (blue) but not in cells from KO^{Adv} (red) or KO^{Wnt} (orange) mice.

(B) Current-voltage (I-V) curve showing that application of the Na_V1.7-specific antagonist PF-771 reduces voltage-gated sodium currents in WT sensory neurons. n=10 cells.

(C) I-V curve showing PF-771 did not markedly affect voltage-gated sodium currents in sensory neurons from Na_V1.7 KO^{Adv} mice. n=7 cells.

(D) I-V curve showing PF-771 did not markedly affect voltage-gated sodium currents in sensory neurons from Na_V1.7 KO^{Wnt} mice. n=9 cells.

Error bars denote standard error of the mean.

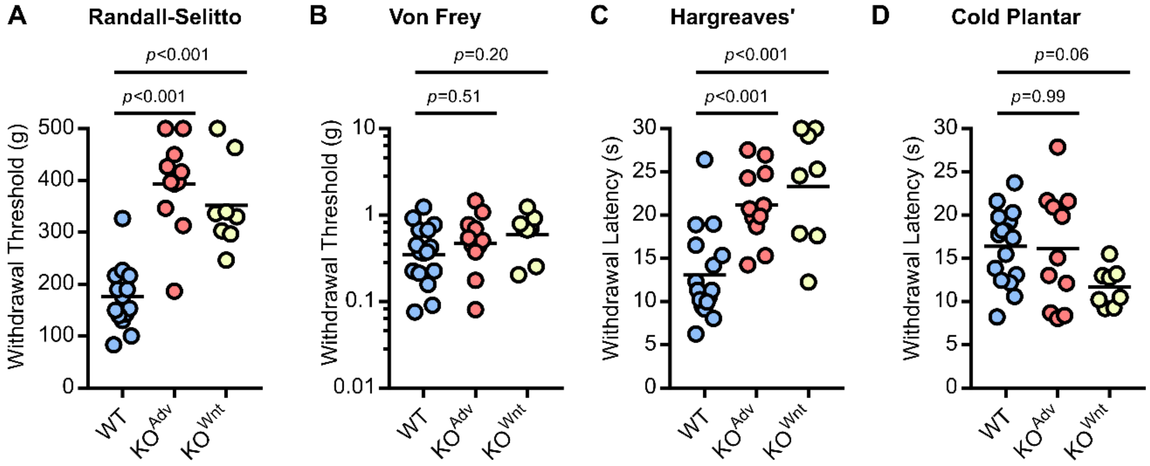
A
Randall-Selitto
B
Von Frey
C
Hargreaves'
D
Cold Plantar
p<0.001
p<0.001
p=0.20
p=0.51
p<0.001
p<0.001
p=0.06
p=0.99
Withdrawal Threshold (g)
Withdrawal Latency (s)
0
100
200
300
400
500
0.01
0.1
1
10
0
5
10
15
20
25
30
WT
KOAdv
KOWnt

Figure S2 (*related to Figure 1*). Peripheral $Na_V1.7$ knockout mice show reduced pain sensitivity.

(A) Both $Na_V1.7$ KO lines show increased tail withdrawal thresholds to noxious mechanical stimuli on the Randall-Selitto test.

(B) $Na_V1.7$ deletion has no effect on withdrawal thresholds to punctate tactile stimuli on the Von Frey test

(C) Both $Na_V1.7$ KO lines show increased withdrawal latencies to radiant heat stimuli on the Hargreaves' test.

(D) $Na_V1.7$ KO^{Adv} mice show unchanged withdrawal latencies to dry ice stimuli on the Cold Plantar test compared to control, however $Na_V1.7$ KO^{Wnt} animals display a small, but not statistically significant, hypersensitivity.

For (A) to (D), means for each KO line were compared to WT control using One-Way ANOVA followed by post-hoc Dunnett's test. n=16 mice for WT, n=11 mice for KO^{Adv} and N=8 mice for KO^{Wnt}.

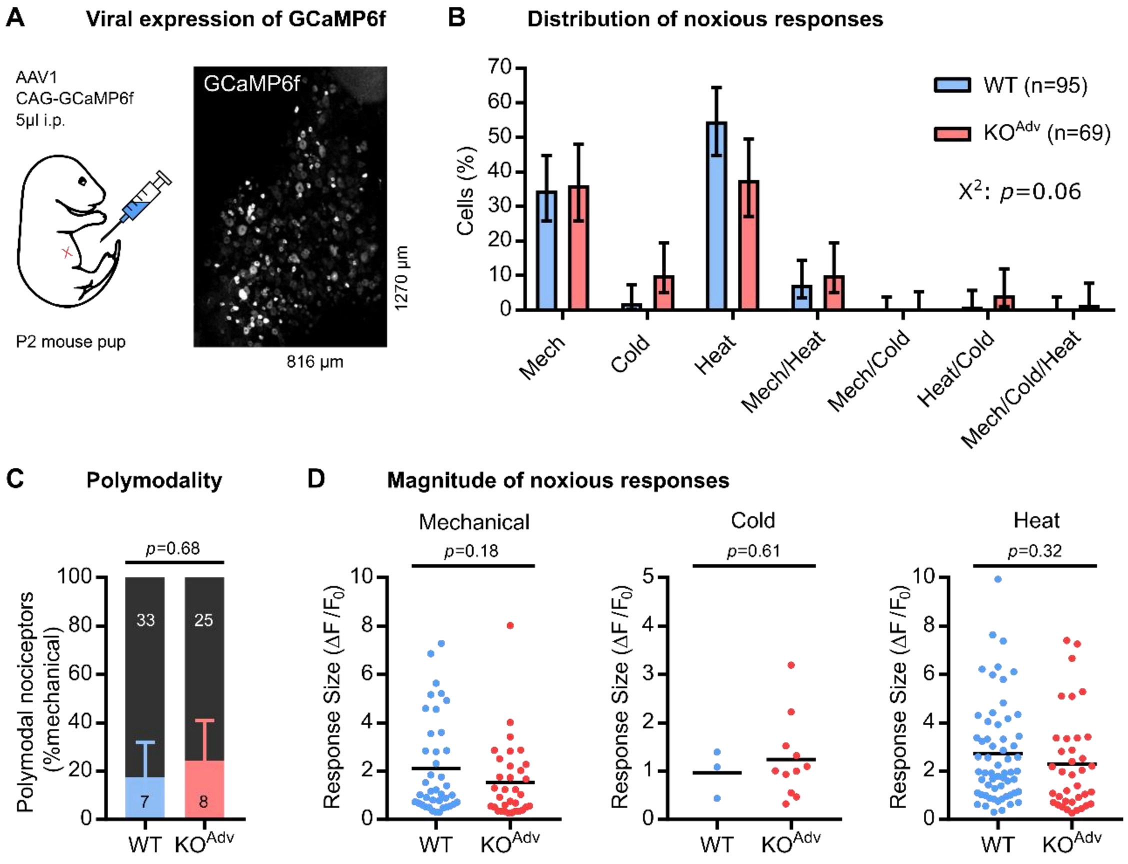

A
Viral expression of GCaMP6f
AAV1
CAG-GCaMP6f
5µl i.p.
P2 mouse pup
GCaMP6f
1270 µm
816 µm
B
Distribution of noxious responses
Cells (%)
WT (n=95)
KO^Adv (n=69)
X^2: p=0.06
Mech
Cold
Heat
Mech/Heat
Mech/Cold
Heat/Cold
Mech/Cold/Heat
C
Polymodality
p=0.68
Polymodal nociceptors (%mechanical)
33
25
7
8
WT
KO^Adv
D
Magnitude of noxious responses
Mechanical
p=0.18
Cold
p=0.61
Heat
p=0.32
Response Size (ΔF/F0)
WT
KO^Adv

Figure S3 (*related to Figure 1*). *In vivo* calcium imaging of Na_V1.7-deficient sensory neurons virally expressing GCaMP6f.

(A) Confocal z-stack of DRG imaged *in vivo* from a Na_V1.7 KO^{Adv} mouse virally expressing GCaMP6f. AAV1-CAG-GCaMP6f was delivered by intraperitoneal injection into P2 mouse pups.

(B) Bar plot summarizing the distribution of all sensory neurons that responded to different noxious stimuli in WT and KO^{Adv} animals. The error bars represent 95% confidence intervals and proportions were compared using Chi-Square test (χ^2). n=95 cells from 4 WT mice (blue) and n=69 cells from 4 KO^{Adv} mice (red). Markedly fewer cells responded to cold in these animals compared to in Pirt-GCaMP3 mice, likely due to biased expression of the virally-delivered GCaMP6f.

(D) Bar plot showing similar prevalence of polymodal nociceptors in WT and KO^{Adv} mice. Polymodal nociceptors are defined as pinch-sensitive neurons that respond to any noxious thermal stimulus (colour) and are expressed as a fraction of mechanically-sensitive cells (black). The error bars represent 95% confidence intervals and proportions were compared using the Chi-Square (χ^2) test with Yates Correction. n=40 cells from WT and n=33 cells from KO^{Adv}.

(E-F) Scatter plots showing similar peak calcium responses ($\Delta F/F_0$) evoked by different noxious stimuli for WT and KO^{Adv}. Mean response magnitude of KO^{Adv} was compared to WT control using an unpaired t test. Mechanical: n=40 cells from WT and n=33 cells from KO^{Adv}. Cold: n=3 cells from WT and n=11 cells from KO^{Adv}. Heat: n=60 cells from WT and n=37 cells from KO^{Adv}.

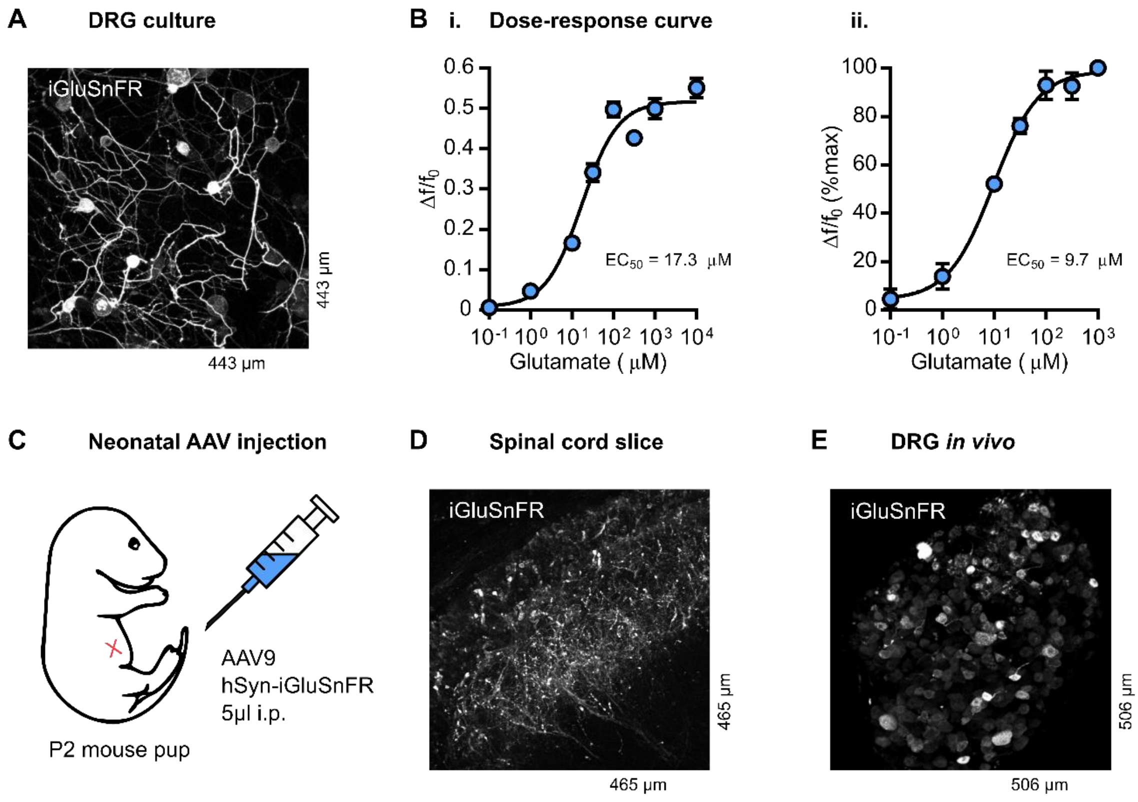

A
DRG culture
iGluSnFR
443 µm
443 µm
B i. Dose-response curve
0.6
0.5
0.4
0.3
0.2
0.1
0
Δf/f0
EC50 = 17.3 µM
10-1 100 101 102 103 104
Glutamate (µM)
ii.
100
80
60
40
20
0
Δf/f0 (%max)
EC50 = 9.7 µM
10-1 100 101 102 103
Glutamate (µM)
C
Neonatal AAV injection
AAV9
hSyn-iGluSnFR
5µl i.p.
P2 mouse pup
D
Spinal cord slice
iGluSnFR
465 µm
465 µm
E
DRG in vivo
iGluSnFR
506 µm
506 µm

Figure S4 (*related to Figure 4*). Expression and function of iGluSnFR in sensory afferents

(A) Confocal z-stack of iGluSnFR-expressing cultured dorsal root ganglia neurons, *in vitro*.

(B) Dose-response curve (i.) of iGluSnFR fluorescence ($\Delta F/F_0$) in cultured DRG neurons against extracellular glutamate concentration. n=36-154 neurons depending on tested concentration. EC_{50}=17.3 μM. r^2=0.426. Dose-response curve (ii.) of normalized iGluSnFR fluorescence (% maximum) in cultured DRG neurons against extracellular glutamate concentration. In this experiment, each cell was exposed to all glutamate concentrations tested. n=45 neurons. EC_{50}=9.7 μM. r^2=0.625.

(C) Schematic of neonatal virus injection.

(D) Two-photon z-stack of iGluSnFR-expressing sensory afferent terminals in dorsal horn of transverse spinal cord slice, *ex vivo*. No spinal cord neuron somata express iGluSnFR.

(E) Confocal z-stack of iGluSnFR-expressing sensory afferent cell bodies in L4 dorsal root ganglion, *in vivo*.

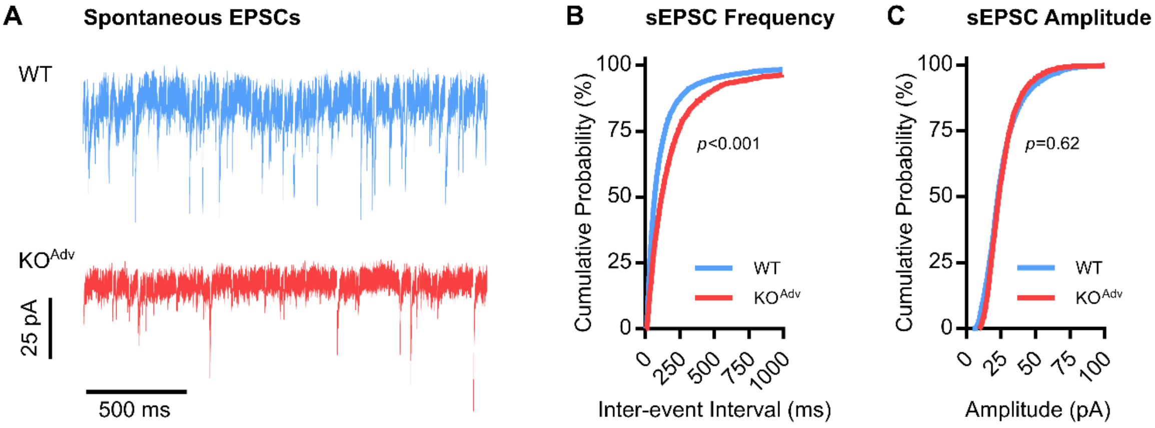
A
Spontaneous EPSCs
WT
KO^Adv
25 pA
500 ms
B
sEPSC Frequency
Cumulative Probability (%)
p<0.001
WT
KO^Adv
Inter-event Interval (ms)
C
sEPSC Amplitude
Cumulative Probability (%)
p=0.62
WT
KO^Adv
Amplitude (pA)

Figure S5 (*related to Figure 4*). Na_V1.7 deletion reduces frequency, but not amplitude, of spontaneous excitatory post-synaptic currents.

(A) Example traces showing spontaneous excitatory post-synaptic currents (sEPSCs) recorded from lamina II neurons in WT and KO^{Adv} mice.

(B) Cumulative probability plots showing sEPSC frequency is reduced in KO^{Adv} animals.

(C) Cumulative probability plots showing sEPSC amplitude is unaltered in KO^{Adv} animals.

Means were compared using unpaired t tests. n=6972 events from 40 WT slices, and n=2406 events from 23 KO^{Adv} slices.

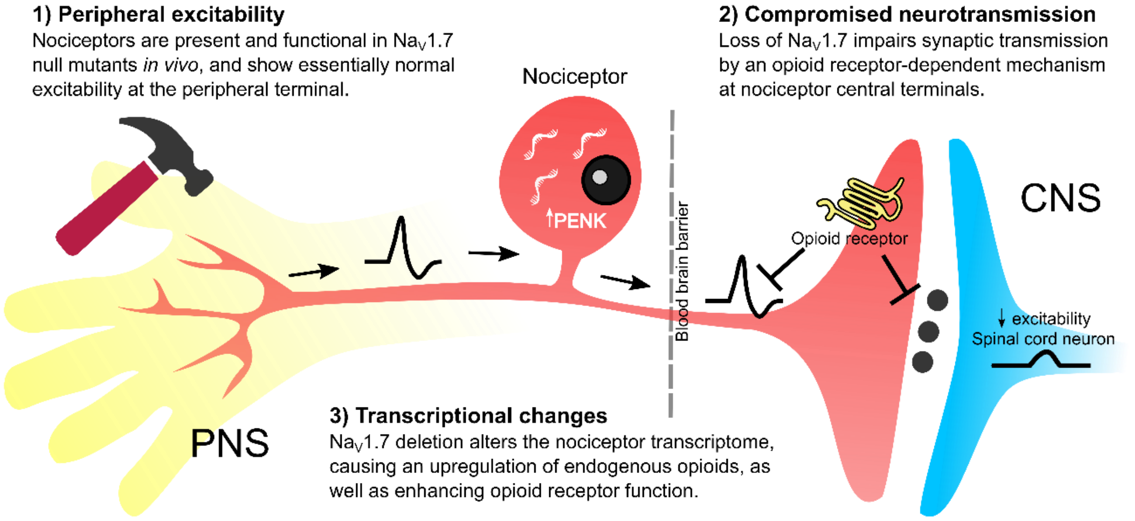

1) Peripheral excitability
Nociceptors are present and functional in $Na_V1.7$ null mutants *in vivo*, and show essentially normal excitability at the peripheral terminal.
Nociceptor
2) Compromised neurotransmission
Loss of $Na_V1.7$ impairs synaptic transmission by an opioid receptor-dependent mechanism at nociceptor central terminals.
↑PENK
Blood brain barrier
Opioid receptor
CNS
↓ excitability
Spinal cord neuron
PNS
3) Transcriptional changes
$Na_V1.7$ deletion alters the nociceptor transcriptome, causing an upregulation of endogenous opioids, as well as enhancing opioid receptor function.

Figure S6 (*related to Figure 8*). Mechanisms of analgesia after loss of $Na_V1.7$.

Cartoon showing the effects of $Na_V1.7$ deletion on nociceptor function.

1) *Peripheral excitability:* Some, but not all, humans with $Na_V1.7$ null mutations show reduced intra-epidermal fibre density, while $Na_V1.7$-deficient sensory neurons are less excitable *in vitro*. But, *in vivo*, terminal excitability is essentially normal, and neurogenic inflammation is preserved. Thus the peripheral terminal is likely not the locus of analgesia.

2) *Compromised neurotransmission:* Synaptic transfer from nociceptor terminals to spinal cord dorsal horn neurons is impaired after loss of $Na_V1.7$. These synaptic deficits depend on opioid receptors, which can suppress both synaptic release and terminal excitability. As reported by Alles *et al* (2020), dorsal horn neurons are also less excitable due to absent post-synaptic $Na_V1.7$.

3) *Transcriptional changes:* Loss of $Na_V1.7$ leads to an upregulation of PENK, encoding pre-preproenkephalin, resulting in increased endogenous opioid signalling. Concomitantly, reduced sodium ingress following deletion of $Na_V1.7$ leads to enhanced opioid receptor function inhibiting neurotransmitter release. Analgesia in mice and humans lacking $Na_V1.7$ is thus dependent on opioids.

PARTICIPANT	MUTATION	CHARACTERIZATION
Male 1	c.377+5C>T (intronic) – point mutation in splice donor site in intron 3 resulting in the use of a cryptic splice donor site, leading to a frameshift and premature stop codon in exon 4 (McDermott et al., 2019; Shaikh et al., 2018).	Pathogenic (Shaikh et al., 2018).
	c.2686C>T (R896W) – amino acid change in exon 16 (McDermott et al., 2019).	No Na_V1.7 current when expressed in HEK293T cells (McDermott et al., 2019).
Male 2	c.2488C>T (R830X) – premature stop codon in exon 16 (Ramirez et al., 2014).	No Na_V1.7 current when expressed in HEK293T cells (McDermott et al., 2019).
	c.5318delA (FS1773) – 1 bp deletion in exon 27, that induces a frameshift at position 1773 in the C terminal domain (Ramirez et al., 2014).	8-fold reduction in Na_V1.7 current density versus WT when expressed in HEK293T cells (McDermott et al., 2019).

Table S1 (*related to Figure 8*). Mutations in *SCN9A* of human participants.

Summary of previous genetic and functional characterization of the SCN9A mutations carried by the compound heterozygous male participants in this study.

PCR PRODUCT	FORWARD PRIMER	REVERSE PRIMER
Pirt-GCaMP3		
Pirt WT (300 b.p.)	TCCCCTCTACTGAGA GCCAG	GGCCCTATCATCCTGAGC AC
GCaMP3 (400 b.p.)	TCCCCTCTACTGAGA GCCAG	ATAGCTCTGACTGCGTGA CC
$Na_V1.7$ flox		
$Na_V1.7$ WT (382 b.p.)	CAGAGATTTCTGCAT TAGAATTTGTTC	GCAAATCATAATTAATTC ATGACACAG
$Na_V1.7$ flox (527 b.p.)	CAGAGATTTCTGCAT TAGAATTTGTTC	GCAAATCATAATTAATTC ATGACACAG or AGTCTTTGTGGCACACGT TACCTC
$Na_V1.7$ KO (395 b.p.)	CAGAGATTTCTGCAT TAGAATTTGTTC	GTTCCTCTCTTTGAATGC TGGGCA
Advillin-Cre		
Advillin WT (480 b.p.)	CCCTGTTCACTGTGA GTAGG	AGTATCTGGTAGGTGCT TCCAG
Cre (180 b.p.)	CCCTGTTCACTGTGA GTAGG	GCGATCCCTGAACATGTC CATC
Wnt1-Cre		
Wnt1-Cre (628 b.p.)	CTCATTGTCTGTGGC CCTGA	AAATGTTGCTGGATAGTT TTTACTGCC
OMP-Cre		
OMP WT	TGGCAACAGCTGTA GCACTT	ACAGAGGCCTTTAGGTT GGC
Cre	CATTTGGGCCAGCTA AACAT	CCCGGCAAAACAGGTAG TTA

Table S2 (*related to Figures 1, 2, 3, 4, 5, 6, 7 and 8*). Primers for mouse line genotyping. Forward and reverse primers required for each PCR used for genotyping.
